# N-terminomics identifies widespread endoproteolysis and novel methionine excision in a genome-reduced bacterial pathogen

**DOI:** 10.1038/s41598-017-11296-9

**Published:** 2017-09-11

**Authors:** Iain J. Berry, Veronica M. Jarocki, Jessica L. Tacchi, Benjamin B. A. Raymond, Michael Widjaja, Matthew P. Padula, Steven P. Djordjevic

**Affiliations:** 1 The ithree institute, University of Technology Sydney, PO Box 123, Broadway, NSW 2007 Australia; 2 Proteomics Core Facility, University of Technology Sydney, PO Box 123, Broadway, NSW 2007 Australia

## Abstract

Proteolytic processing alters protein function. Here we present the first systems-wide analysis of endoproteolysis in the genome-reduced pathogen *Mycoplasma hyopneumoniae*. 669 N-terminal peptides from 164 proteins were identified, demonstrating that functionally diverse proteins are processed, more than half of which 75 (53%) were accessible on the cell surface. Multiple cleavage sites were characterised, but cleavage with arginine in P1 predominated. Putative functions for a subset of cleaved fragments were assigned by affinity chromatography using heparin, actin, plasminogen and fibronectin as bait. Binding affinity was correlated with the number of cleavages in a protein, indicating that novel binding motifs are exposed, and protein disorder increases, after a cleavage event. Glyceraldehyde 3-phosphate dehydrogenase was used as a model protein to demonstrate this. We define the rules governing methionine excision, show that several aminopeptidases are involved, and propose that through processing, genome-reduced organisms can expand protein function.

## Introduction

Terminomics is the study of the terminal amino acid sequences in mature proteins. The process is achieved typically by sequencing N-terminal and, to a lesser extent, C-terminal peptides^1^. Terminomics is a rapidly expanding discipline in protein science which was spearheaded initially by developments in mass spectrometry, and the need to understand proteolytic systems that influence complex biological processes such as inflammation, malignancy and wound healing^2^. The number of sequenced genomes is expanding at a rapid rate and increasingly, we are reliant on gene prediction algorithms to identify open reading frames (ORFs). Gene prediction algorithms continue to improve, but are far from perfect, even for eukaryotes where translation initiation is quite strict. Translation initiation in prokaryotes can be flexible, and this often presents challenges for bioinformatics^1^.

N-terminomic and proteogenomic studies of prokaryotes are still in their infancy but have the potential to reveal novel proteolytic processes that determine the identity and existence of mature proteoforms^3–5^. Terminomics has informed bioinformatics of the quality of predictive gene annotation by generating biochemical evidence for the boundaries of gene sequences that define a functional ORF^1^ and can shed light on the activity and specificity of proteases, and how they impact the proteome. Proteases regulate a myriad of physiological processes by exerting control over protein function, localisation, and turnover^2, 6^. The removal of the initiating methionine (iMet) is a ubiquitous proteolytic process known as N-terminal methionine excision (NME). Methionine initiates protein synthesis for essentially all eukaryotic and prokaryotic proteins^4^. Depending on the organism and stage of cellular growth, 50–70% of proteins are a target of NME^7, 8^. In bacteria, the NME process is possible through the combined actions of peptide deformylase (PDF) and methionine aminopeptidase (MAP). PDF removes N-formyl groups from the iMet residue allowing MAP to remove the iMet. The efficiency of the cleavage reaction is influenced in part by the precise sequence of amino acids that follow the iMet residue. Methionine removal typically occurs where the next amino acid, located in the P1′ position, is small and uncharged^9^. Studying NME has been challenging as MAP gene inactivation is lethal in many model organisms including *Salmonella enterica* serovar Typhimurium^10^, *Escherichia coli* ^11^ and *Saccharomyces cerevisiae* ^12^. NME plays an important role in directing subcellular localisation of proteins, and influences posttranslational modification events (PTMs), protein folding and activity^13^.

Terminomics enables an analysis of post-translational maturation processes performed by proteases as they shape the functional, mature proteome^3^. Pathogenic bacteria, such as *Mycobacterium tuberculosis* and *Chlamydia trachomatis*, proteolytically process virulence proteins as part of an overall mechanism to activate them^14–17^. Understanding the role and scope these processing events play in prokaryotes, is critical in combatting infectious diseases. In an effort to characterise the role of proteolytic processing in shaping a bacterial proteome, we selected *Mycoplasma hyopneumoniae*, a pathogen that incurrs large losses to swine production globally^18^, contributes significantly to the antimicrobial load used in swine production^19–22^ and because protein processing and proteases feature prominently in its armoury of virulence attributes^23–35^. Furthermore, *M*. *hyopneumoniae* is a genome-reduced pathogen (the genome of the type strain J comprises 897,405 bp, and 671 predicted protein coding sequences) that is reliant on degradative enzymatic pathways to sequester amino acids, lipids and nucleotides from its only known host, the pig^36^.

The extent of proteolytic processing in *M*. *hyopneumoniae* is unknown, but it has been shown to process lipoproteins^35^, surface moonlighting proteins^35, 37^ and, most extensively, adhesins such as MHJ_0494 (P159)^24, 32^ and those belonging to the P97 and P102 families^23, 25, 26, 30, 33, 34, 38^. Adherence to the ciliated epithelium is pivotal to colonization and is in part mediated by the P97 and P102 adhesin families and other adhesins that directly interact with the host extracellular matrix (ECM), via glycosaminoglycans (GAGs)^24, 27, 28, 31, 38–40^, fibronectin^26, 28, 29, 38^, and plasminogen^26, 28, 29, 31, 41^. Notably, a glutamyl aminopeptidase (GAP; MHJ_0125) and a leucine aminopeptidase (LAP; MHJ_0461) that perform alternative functions on the cell surface (known as protein moonlighting^42, 43^), also function as accessory adhesins^37, 40^. Cleavage motifs with the sequence S/T-X-F↓X-D/E, and L-X-V↓X-V/A-X have been previously characterised in these adhesin families^23, 27, 30^. As such, protein cleavage provides *M*. *hyopneumoniae* with a mechanism to manipulate its cell surface protein topography in a manner that is akin to ectodomain shedding in eukaryotes^32, 33^. These processing events may influence Pathogen-Associated Molecular Patterns (PAMPs), profoundly altering the presentation of virulence factors to host cell receptors and immune effectors^44^ while generating peptide fragments with new functions.

Recently we analyzed the proteome of *M*. *hyopneumoniae* using protein-centric approaches such as one- and two-dimensional GeLC-MS/MS to expand our understanding of global processing events^35^. Here we determine the extent of proteolytic processing in *M*. *hyopneumoniae* by characterising the N-terminome using charge-reversal enrichment of N-terminal peptides and LC-MS/MS. Notably, we identified a large number of N-terminally labeled peptides internal to the protein ORF and several proteins that underwent NME when unexpected amino acids occupied the P1′ position. To explore this further, we purified several aminopeptidases as polyhistidine fusion proteins, including a putative MAP (MHJ_0169). The enzymatic activity of each of the recombinant proteases was investigated using a series of synthesised peptides with amino acids carrying different physical and electrostatic properties in the P1′ position. We also constructed a comprehensive database of proteins and their cleavage products and employed two complementary, orthogonal approaches to characterise cell surface proteins of *M*. *hyopneumoniae* to identify molecules that are both proteolytically processed and accessible on the bacterial cell surface. Affinity chromatography columns containing heparin, actin, fibronectin or plasminogen as bait were employed as enrichment strategies to explore the functional repertoire of cell surface proteoforms.

## Results

### N-terminal sequences of *M*. *hyopneumoniae*

In this study, N-terminal peptides were enriched by charge based separation of internal and C-terminal peptides and sequenced by LC-MS/MS. This approach yielded 669 unique N-terminal peptide sequences which aligned to 164 predicted protein ORFs (Supplementary Data S1). These data constitute ~25% of the predicted proteome of *M*. *hyopneumoniae* (strain J) and is one of the most comprehensive microbial N-terminome datasets currently available. Of the 669 peptides, 81 were N-terminal peptide sequences that commenced from the iMet of 81 protein ORFs (Supplementary Data S2), as predicted from the genome sequence of *M*. *hyopneumoniae* strain J. These data confirm that the computationally-predicted starts sites for these 81 proteins are accurate.

We identified N-terminal sequences of 47 proteins where the iMet was removed from the N-terminus of the nascent polypeptide chain (Supplementary Data S3). Of the 47 proteins, 35 conformed to NME rules^8, 45^ with the iMet most frequently removed when serine (S) or alanine (A) occupied the P1′ position (Table 1).

**Table 1:** Sequence analysis of iMet removed N-terminal peptides.

P1′ Residue		P2′ Residue	
S	16	K	16
A	14	N	9
T	4	I	4
*N	4	Y	4
*K	3	A	3
*D	1	L	3
*F	1	Q	3
G	1	R	2
*L	1	S	2
V	1	V	1
*Q	1		

Distribution of amino acids in the P1′ and P2′ positions of iMet removed N-terminal peptides. *Denotes amino acids that deviate from typical NME.

Notably, we found several deviations from standard NME patterns. Firstly, 12 proteins showed evidence of methionine removal but contained amino acids in the P1′ position that do not conform to NME rules (indicated in Table 1). These proteins had asparagine (4 proteins), lysine (3 proteins), aspartic acid, phenylalanine, leucine, valine and glutamine (1 protein each) in the P1′ position. Second, a subset of thirteen (13) iMet removed proteins also appeared in the list of proteins with iMet retained at the N-terminus, suggesting that both proteoforms are generated (Table 2). Interestingly, BLAST searches of Uniprot and NCBI databases failed to identify any peptide deformylase (PDF) protein in *M*. *hyopneumoniae* and many other species of Mycoplasmas. Of the 171 Mycoplasma proteomes stored in the Uniprot database, only 65 contained putative annotations for PDFs (Supplementary Data S4).

**Table 2:** N-terminal dimethylated peptides detected with both iMet present and with iMet removed.

Gene names	Protein names	Sequence
MHJ_0127	50S ribosomal protein L21	(M)FAIIKTGGR
MHJ_0078	NADH oxidase	(M)KIISIGTNHAGTSFLR
MHJ_0365	Putative ABC transporter permease protein	(M)KKDNENLDYQNADFEYEIKKIR
MHJ_0667	Transcription elongation factor GreA	(M)KNIVDDKILLTQQKLEEIEKELEHLINVER
MHJ_0189	50S ribosomal protein L4	(M)NKISEISIQSQKTENLVKFNANDDL PKSLFEQKEPHFQAIFDSILSER
MHJ_0029	DNA topoisomerase (ATP-hydrolyzing)	(M)NKSLDSVINSQLEKILAEKFIR
MHJ_0181	30S ribosomal protein S17	(M)NNLTLEKKAQTR
MHJ_0650	30S ribosomal protein S9	(M)NQPELSYYGTGR
MHJ_0125	Putative aminopeptidase	(M)SILEKMKKYCDIDGMSR
MHJ_0080	Purine-nucleoside phosphorylase	(M)TAHIEAKKNEIAPIVLMPGDPLR
MHJ_0506	Phosphate acetyltransferase	(M)TYQEYLQAR
MHJ_0110	Adenine phosphoribosyltransferase	(M)QINLEKYIR
MHJ_0611	Phosphocarrier protein HPr	(M)VSFSAVVIDKLGFHAR

We interrogated the remaining N-terminal data for any sequences which represent possible alternative translation initiation sites (aTIS) or incorrectly annotated ORFs. We identified sequences with methionine as the first residue or a methionine predicted before the N-terminal peptide within 50 amino acids of the predicted protein ORF. This produced a list of 9 N-terminal peptides from 7 proteins shown in Supplementary Data S5. For 30S ribosomal protein S17 (MHJ_0181) and LicA (MHJ_0036) we observed N-terminal peptides with both the methionine present and removed at positions 1, 2, 4 and 5; and 1, 30 and 31 respectively suggesting that these sites are produced by amino/endopeptidases and not likely to be *bona fide* alternative translation sites. Upstream N-terminal peptides beginning from position 1 or 2 were also detected for 5 of the 7 proteins in this list (indicated in Supplementary Data S5). However, for 30S ribosomal protein S16 (MHJ_0278) and guanylate kinase (MHJ_0149), no upstream N-terminal peptide could be found in our data set, and may either represent the correct translational start sites for these proteins or the product of N-terminal proteolytic processing and maturation.

### Characterizing putative *M*. *hyopneumoniae* methionine aminopeptidase (MAP)

**Figure 1:**
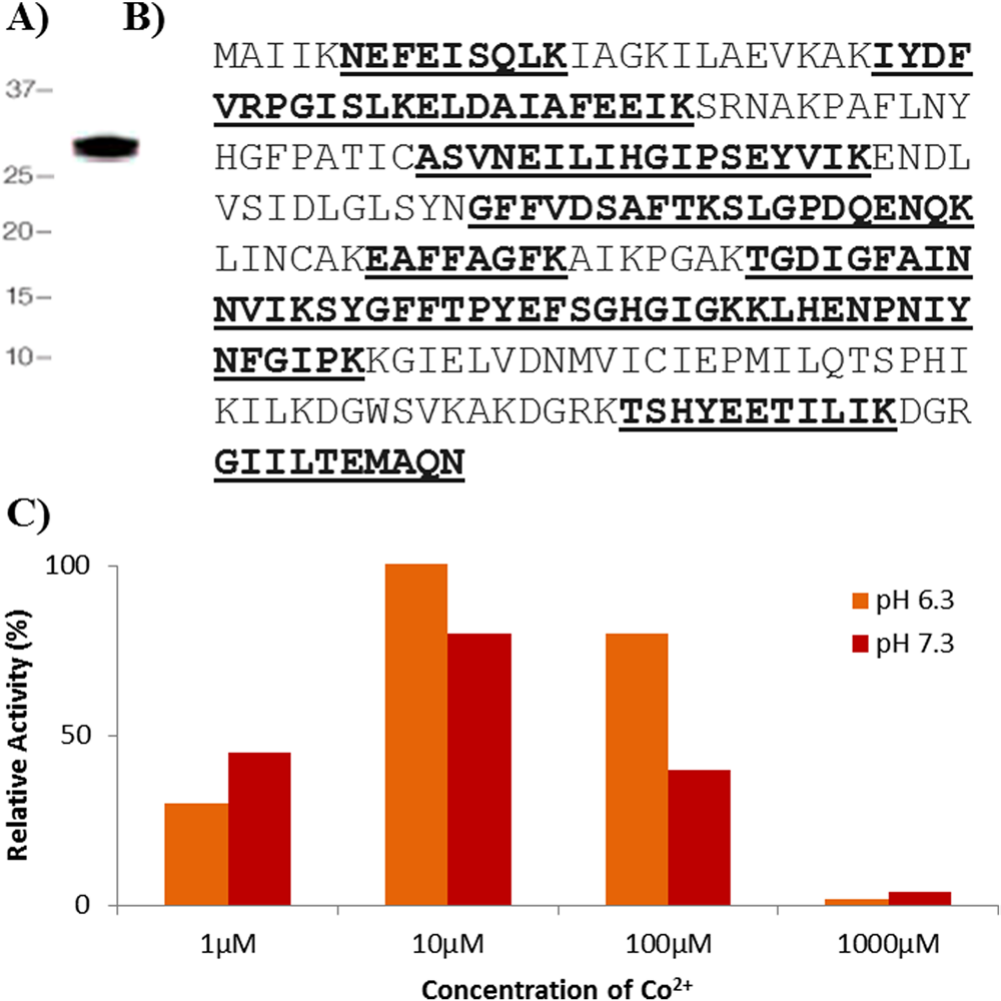
rMHJ_0169 resolves as a monomer of its theoretical mass and shows activity with Co^2+^. (**A**) rMHJ_0169 resolved as a single band at ~30 kDa after SDS-PAGE. (**B**) Tryptic peptides identified by LC-MS/MS peptide map to MHJ_0619 (underlined and bold) and comprise 61% sequence coverage. (**C**) rMHJ_0169 was most efficient at cleaving Met-AMC at pH 6.3 and in the presence of 10 µM of Co^2+^.

*M*. *hyopneumoniae* MAP (rMHJ_0169) was expressed as a polyhistidine fusion protein in *E*. *coli* and purified under native conditions using Ni^2+^ affinity chromatography. rMHJ_0169 migrated as a monomer of ~30 kDa (Fig. 1A) during SDS-PAGE, consistent with the protein’s theoretical mass of 28 kDa. Tryptic peptides generated by digesting the protein in the ~30 kDa band followed by LC-MS/MS mapped to MHJ_0169 (Fig. 1B), confirming its identity.

rMHJ_0169 was incubated against a range of AMC-coupled amino acid substrates and various divalent cation cofactors over a pH range from 5.5–8.8 using a standard kinetic assay. rMHJ_0169 was only active against Met-AMC, and only in the presence of Co^2+^. Evidence of activation began at 1 µM of Co^2+^, was most efficient at 10 µM, and became inhibitory at concentrations higher than 1000 µM. rMHJ_0169 was most active at pH 6.3 followed by pH 7.3 (Fig. 1C) and had no activity at pH 5.5 and pH 8.8.

### Multiple aminopeptidases are involved in NME in *M*. *hyopneumoniae*

**Figure 2:**
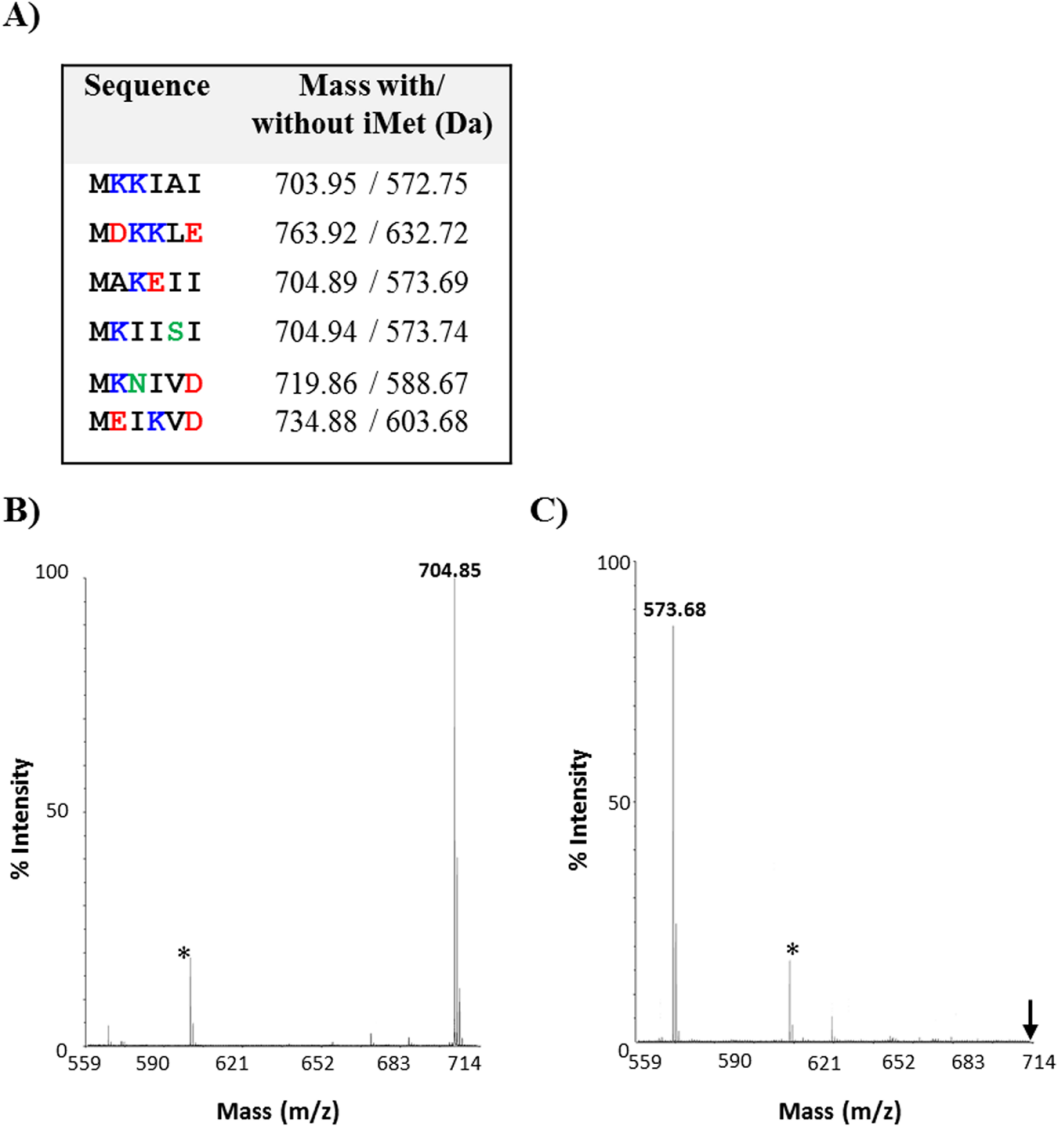
Amino acid sequences of peptide substrates and MALDI spectra of MAP activity against MAKEII peptide. (**A**) Synthetic peptide sequences and their masses with and without N-terminal methionine (iMet). (**B**) Control MALDI-TOF MS/MS spectra of MAKEII in the absence rMHJ_0169. A prominent peak was seen at 704.85 Da which equates to the peptides intact mass. (**C**) MALDI-TOF MS/MS spectra of MAKEII after 60 mins incubation with rMHJ_0169 and Co^2+^ cofactor at pH 7.3. Parent peak was absent (↓) and a new, prominent peak was seen at 573.68 Da, the mass of AKEII, indicating the removal of M by rMHJ_0169. *denotes a matrix artifact observed in both control and test.

To determine the specificity of rMHJ_0169 for removing iMet, we constructed a panel of six hexapeptides selected from our N-terminal peptide dataset (Supplementary Data S2 and S3) that commence with their iMet. Each hexapeptide contained a different combination of amino acids in the P1′ (K, D, A, E) and P2′ (K, I, N) position (Fig. 2A). rMHJ_0169 was incubated for 60 minutes at 37 °C with each peptide in the presence of Co^2+^ at pH 7.3. Products of the reaction were then analysed by MALDI-TOF MS/MS. rMHJ_0169 was not active against peptides with any amino acid other than alanine at the P1′ position (Fig. 2B and C).

**Figure 3:**
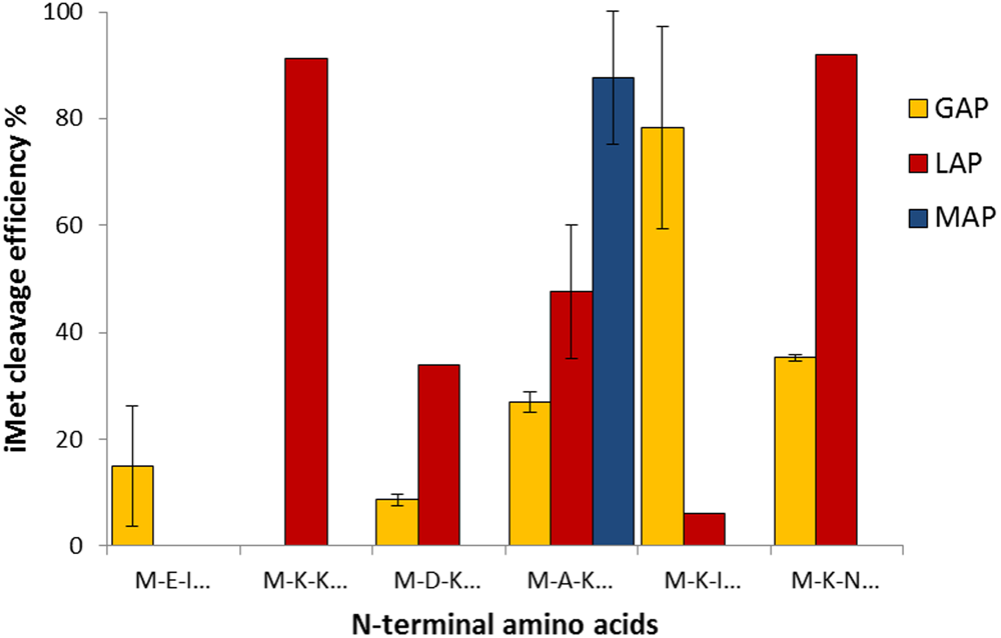
Comparison of iMet removal from peptides by GAP, LAP, and MAP. MAP was most efficient at removing iMet from MAKEII but had no activity against other tested peptides. LAP aminopeptidase activity was strongest in the order MKNIVD < MKKIAI < MAKEII < MKIISI. GAP aminopeptidase activity was strongest in the order MKIISI < MKNIVD < MAKEII < MEIKVD < MDKKLE. MEIKVD and MDKKLE were poor substrates for all tested aminopeptidases.

The peptides were then tested against two previously characterized recombinant aminopeptidases from *M hyopneumoniae*; glutamyl aminopeptidase (GAP: rMHJ_0125)^37^ and leucine aminopeptidase (LAP: rMHJ_0461)^40^. Like MAP, both GAP and LAP were incubated with each substrate for 60 minutes at pH 7.3 and analyzed by MALDI-TOF MS/MS. LAP assays used cofactor Mn^2+^ rather than Co^2+^ as it was previously shown to be the most efficient metal ion cofactor^40^. The best substrates for LAP were MKNIVD (92%) and MKKIAI (91.25%), with some activity against MAKEII (48%) and MDKKLE (34%). LAP showed poor activity against MKIISI (6%), and no activity against MEIKVD. In contrast, GAP was most efficient at cleaving MKIISI (78%) followed by MKNIVD (35%), MAKEII (27%), MEIKVD (15%) and MDKKLE (9%). Unlike LAP, GAP did not cleave MKKIAI. MAP was the most active aminopeptidase for cleaving MAKEII (88%) (Fig. 3).

### The surfaceome of *M*. *hyopneumoniae*

We identified 669 unique N-terminal peptide sequences that mapped to 164 predicted *M*. *hyopneumoniae* proteins (Supplementary Data S1). While a subset of these 164 proteins were known to have a cell surface location, most have distinct functions within the cell and are not predicted to be secreted onto the cell surface. Endoproteolytic events would be expected to adversely impact canonical function of proteins in the cytosol. To investigate this hypothesis further, we characterised the surfaceome of *M*. *hyopneumoniae* using two complementary, orthogonal strategies and identified 159 putative cell-surface proteins (Supplementary Data S6). The composition of these 159 proteins included membrane transporters, lipoproteins, adhesins such as P159 (MHJ_494) and members of the P97 and P102 families, and other membrane proteins. SignalP identified the presence of a classical signal peptide in 20/159 (~13%) proteins. Only 17/159 (~11%) were predicted to be localised onto the cell surface by PSortB. However SecretomeP found that 70/159 (~44%) were either classically or non-classically secreted, revising the number of predicted intracellular proteins down from 98 to 43 (27%). Seven of these predicted intracellular proteins were found by both methods, while the remaining 36 were reproducibly found by one of the methods in replicate experiments.

### Endoproteolytic processing and putative functions of *M*. *hyopneumoniae* surface proteins

The remaining 523 dimethyl labelled N-terminal peptides map to 141 protein sequences that commence downstream of the iMet start site. These neo-N-termini are listed in Supplementary Data S7. The 141 proteins represent ~21% of the predicted proteome of *M*. *hyopneumoniae*. Eighty-two proteins contained two or more N-terminal peptides, indicating a complex processing mechanism. It is notable that 75 proteins (53%) identified on the surface of *M*. *hyopneumoniae* overlap with those that are a target of multiple internal processing events (see Table 3). Table 4 tallies the amino acids surrounding each scissile bond (P4-P3-P2-P1↓P1′-P2′-P3′-P4′) in these 75 proteins.

**Table 3:** *M*. *hyopneumoniae* surface proteins, found with N-terminally labelled peptides, with bioinformatic analyses and affinity chromatography results.

Gene names	Protein names	N-term	NME	Proteolytic Cleavages	Cytosolic Prediction	TMD	Hep	Act	Fib	Pla
**Metabolic Protein**										
MHJ_0133	L-lactate dehydrogenase	✓		7	✓	✓	✓	✓	✓	✓
MHJ_0506	Phosphate acetyltransferase	✓	✓	4	✓	✓	✓			✓
MHJ_0505	Acetate kinase			1	✓		✓	✓		✓
MHJ_0503	Dihydrolipoamide acetyltransferase		✓	5	✓	✓	✓		✓	✓
MHJ_0487	Phosphoglycerate kinase	✓		5	✓		✓	✓		✓
MHJ_0426	Transketolase	✓		2	✓		✓			✓
MHJ_0349	HIT-like protein	✓		1	✓		✓			✓
MHJ_0242	Enolase		✓	6	✓	✓	✓	✓	✓	✓
MHJ_0157	Phosphopentomutase	✓		1	✓		✓			
MHJ_0122	Pyruvate kinase	✓		5	✓	✓	✓	✓		✓
MHJ_0112	Pyruvate dehydrogenase			13	✓	✓	✓	✓	✓	✓
MHJ_0111	Pyruvate dehydrogenase E1-alpha subunit			12	✓	✓	✓		✓	✓
MHJ_0107	ATP-dependent 6-phosphofructokinase		✓	1	✓	✓	✓	✓		✓
MHJ_0031	Glyceraldehyde-3-phosphate dehydrogenase	✓		4	✓		✓	✓	✓	✓
MHJ_0145	Trigger factor			3	✓		✓			✓
MHJ_0631	Putative 5’-nucleotidase			1		✓	✓		✓	✓
MHJ_0071	Adenine phosphoribosyltransferase	✓	✓	4	✓	✓	✓	✓	✓	✓
MHJ_0436	3-hexulose-6-phosphate synthase			1	✓					
MHJ_0078	NADH oxidase	✓		7	✓		✓	✓	✓	✓
**Lipoprotein**										
MHJ_0511	46 kDa surface antigen			3		✓	✓	✓	✓	✓
MHJ_0656	Putative prolipoprotein p65			2		✓	✓	✓	✓	✓
MHJ_0606	ABC transporter xylose-binding lipoprotein			2		✓	✓	✓	✓	✓
MHJ_0363	Putative lipoprotein			4		✓		✓	✓	✓
MHJ_0362	Putative lipoprotein			1		✓	✓	✓	✓	✓
**Adhesin**										
MHJ_0663	Putative adhesin like-protein P146			17		✓	✓	✓	✓	✓
MHJ_0662	Mhp683 homologue	✓		22		✓	✓	✓	✓	✓
MHJ_0493	Putative P216 surface protein	✓		44		✓	✓	✓		✓
MHJ_0195	P102			1	NC	✓	✓	✓	✓	✓
MHJ_0194	P97/P123			13	NC	✓	✓	✓	✓	✓
MHJ_0494	Putative p76 membrane protein			28	NC	✓	✓	✓	✓	✓
**Protease**										
MHJ_0659	XAA-PRO aminopeptidase			2	✓		✓			
MHJ_0525	Lon protease			4	✓	✓	✓			✓
MHJ_0522	Oligoendopeptidase F	✓		3	✓		✓			
MHJ_0461	Leucine aminopeptidase	✓		2	✓		✓			✓
MHJ_0202	ATP-dependent zinc metalloprotease FtsH			5		✓	✓		✓	✓
MHJ_0125	Glutamyl aminopeptidase	✓		8	✓		✓			✓
**Ribosomal**										
MHJ_0577	30S ribosomal protein S4			9	✓			✓	✓	✓
MHJ_0287	30S ribosomal protein S6			6	NC			✓	✓	✓
MHJ_0190	50S ribosomal protein L3			6	NC			✓		✓
MHJ_0187	50S ribosomal protein L2		✓	8	NC			✓		✓
MHJ_0183	50S ribosomal protein L16	✓		1	✓			✓	✓	✓
MHJ_0179	50S ribosomal protein L24			3	NC		✓	✓		✓
MHJ_0176	30S ribosomal protein S8			1	✓			✓		✓
MHJ_0175	50S ribosomal protein L6			4	✓			✓		✓
MHJ_0165	30S ribosomal protein S11			1	NC			✓	✓	✓
MHJ_0128	50S ribosomal protein L27			3	NC		✓	✓		✓
MHJ_0127	50S ribosomal protein L21	✓	✓	1	✓		✓	✓		✓
MHJ_0051	30S ribosomal protein S2			1	NC			✓	✓	✓
**Translation/Transcription**										
MHJ_0535	Ribosome recycling factor			1	✓		✓			
MHJ_0667	Transcription elongation factor GreA	✓	✓	6	✓					
MHJ_0618	DNA-directed RNA polymerase subunit beta	✓		3	✓	✓	✓			✓
MHJ_0617	DNA-directed RNA polymerase subunit beta			4	✓	✓	✓			
MHJ_0067	Bacterial nucleoid DNA-binding protein			1	NC			✓		✓
**Transporter**										
MHJ_0611	Phosphocarrier protein HPr	✓	✓	1	✓					
MHJ_0469	Phosphoenolpyruvate-protein phosphotransferase			2	✓	✓				✓
**Membrane**										
MHJ_0227	Periplasmic sugar-binding protein			1	NC	✓	✓		✓	✓
**Protein Folding**										
MHJ_0524	Elongation factor Tu		✓	26	✓	✓	✓	✓	✓	✓
MHJ_0071	Elongation factor G			3	✓	✓				
MHJ_0064	Chaperone protein DnaJ		✓	3	NC	✓		✓		✓
MHJ_0063	Chaperone protein DnaK		✓	20	✓	✓	✓	✓	✓	✓
MHJ_0052	Elongation factor Ts		✓	1	✓		✓			
**Redox**										
MHJ_0504	Dihydrolipoyl dehydrogenase	✓		5	✓	✓	✓		✓	✓
MHJ_0380	Putative thioredoxin			4	✓		✓			
MHJ_0228	Putative myo-inositol 2-dehydrogenase	✓		1	NC		✓			
MHJ_0222	Putative myo-inositol 2-dehydrogenase	✓		3	✓		✓			
MHJ_0219	Putative methylmalonate-semialdehyde dehydrogenase			3	✓	✓	✓			
**Uncharacterised**										
MHJ_0486	Uncharacterized protein			2	✓	✓	✓			
MHJ_0373	Uncharacterized protein			3	✓		✓			
MHJ_0369	Uncharacterized protein			2	NC	✓	✓	✓		✓
MHJ_0326	Uncharacterized protein		✓	5	NC	✓	✓		✓	✓
MHJ_0212	Uncharacterized protein			10	NC	✓	✓	✓		✓
MHJ_0009	Uncharacterized protein			3	✓	✓	✓			

*N-term column indicates sequences detected beginning at the predicted Open Reading Frame starting with the iMet residue. NME indicates sequences were detected with the initiating methionine removed. Proteolytic Cleavage column indicates the number of N-terminal peptides mapped to sequences starting from any other position in the protein. Cytosolic protein prediction made by PSORTb, with mention made to proteins secreted by non-classical (NC) mechanisms predicted by SecretomeP. Presence of transmembrane domains (TMDs) was predicted by TMPred. Final four columns indicate whether the protein was found in heparin (Hep), actin (Act), fibronectin (Fib) and/or plasminogen (Pla) affinity chromatography elutions.

**Table 4:** Specificity matrix of cleavage events present in surface exposed *M*. *hyopneumoniae* proteins.

Amino acid	P4	P3	P2	P1	P1′	P2′	P3′	P4′
Glycine (G)	18	23	22	8	18	19	19	12
Alanine (A)	24	23	32	3	19	32	26	24
Proline (P)	13	12	22	4	0	20	18	22
Valine (V)	21	17	23	6	23	19	20	24
Leucine (L)	35	35	31	4	25	36	38	28
Isoleucine (I)	26	32	27	1	21	31	20	24
Methionine (M)	10	6	2	1	4	7	3	2
Phenylalanine (F)	16	17	12	8	10	14	20	12
Tyrosine (Y)	5	11	9	1	13	16	10	8
Tryptophan (W)	1	4	4	2	2	2	1	3
Serine (S)	1	28	19	9	**32**	11	16	25
Threonine (T)	20	16	18	0	**43**	15	18	26
Asparagine (N)	21	19	18	30	24	18	24	21
Glutamine (Q)	13	20	23	7	19	18	15	19
Aspartic Acid (D)	21	17	21	2	6	9	20	14
Glutamic Acid (E)	29	15	13	3	12	15	21	29
Lysine (K)	31	32	25	11	**42**	37	36	32
Arginine (R)	15	3	6	**228**	8	10	4	1
Histidine (H)	1	3	5	3	12	4	3	7

**Figure 4:**
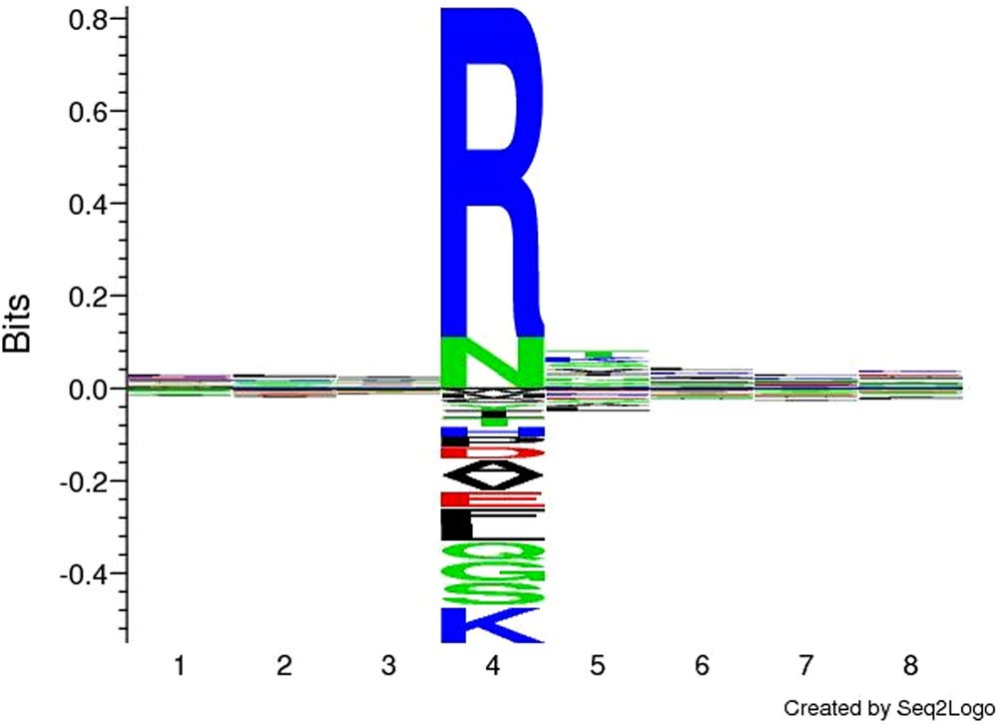
Cleavage site sequence logo for surface exposed *M*. *hyopneumoniae* proteins. The most prevalent proteolytic activity appears to be non-specific trypsin-like cleavages at arginine (R).

The most common cleavage event occurred when arginine (R) was in the P1 position irrespective of the amino acid in position P1′ except proline (P) (Fig. 4). This cleavage behaviour is indicative of trypsin-like activity; however, *M*. *hyopneumoniae* has no annotated trypsin-like proteases. A search for conserved domains within 270 uncharacterised *M*. *hyopneumoniae* proteins revealed that MHJ_0568 possesses a trypsin-like peptidase domain between amino acids 63–190 (E-value 6.62e^−23^). This quest also uncovered two additional putative subtilisin-like proteases (MHJ_0398 and MHJ_0332) and another putative glutamyl aminopeptidase (MHJ_0496) (Supplementary Data S8). Thus there are more potential proteases than originally described in the genome sequences of this species^36, 46^ suggesting that protein processing/catalysis is a major feature of this genome-reduced pathogen.

As the remaining cleavages at hydrophobic, nucleophilic, amide and acidic residues are likely due to several different proteases, specific cleavage motifs to specific proteases could not be assigned with confidence. However, cleavage motifs identified in previous studies were verified using N-terminomics. The motif L-X-V↓X-V/A-X was found in P97 paralogue MHJ_0369 at ^761^L-n-V↓a-V-s^766^, and the S/T-X-F↓X-D/E motif was found in P123 homologue MHJ_0194 at ^192^T-n-F↓a-D^196^ and in the Mhp683 homologue at ^343^T-e-F↓v-E^347^. Interestingly, many proteins underwent proteolysis when a nucleophilic residue (S/T) was in the P1′ position (Table 4).

**Figure 5:**
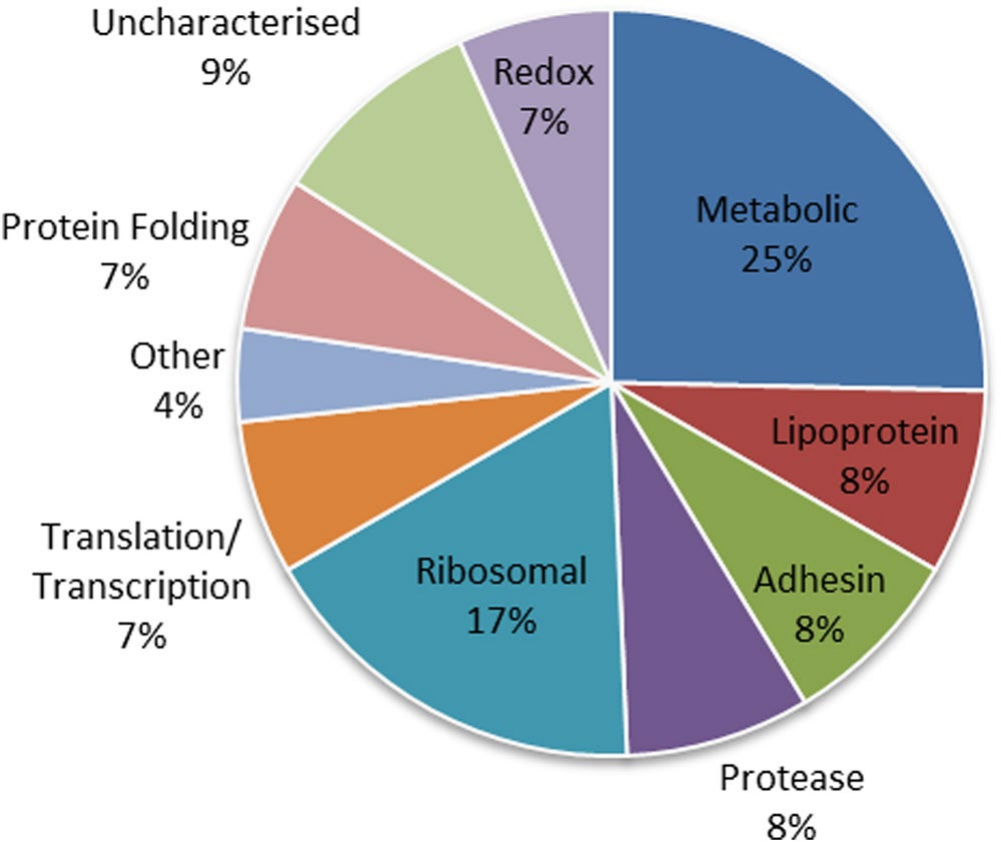
Predicted functions of endoproteolytically cleaved surface proteins. The most common category of endoproteolytically cleaved proteins found on the surface of *M*. *hyopneumoniae* were metabolic (25%) and ribosomal proteins (17%), followed by those listed as uncharacterised (9%).

**Figure 6:**
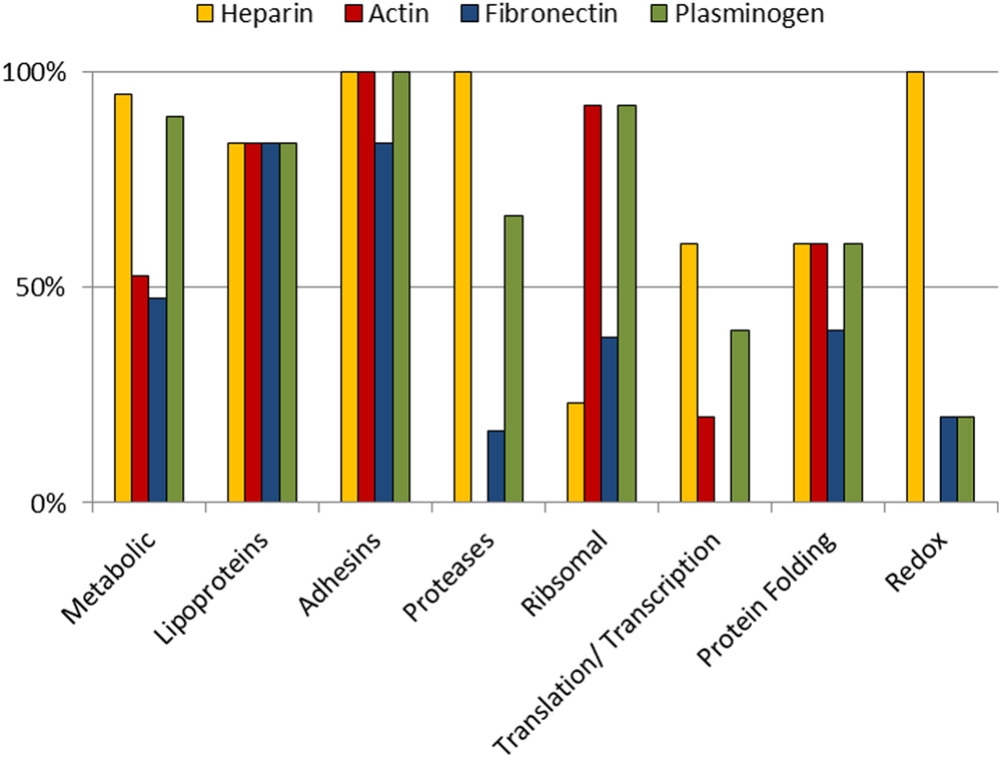
Number of cleaved surface proteins within functional groups identified in affinity chromatography columns. Adhesins and lipoproteins were most frequently recovered from columns coupled with heparin, actin, fibronectin and plasminogen. Almost all metabolic proteins were retained during heparin-agarose chromatography and on columns coupled with plasminogen, but only ~50% were retained on columns coupled with actin and fibronectin. All proteases bound heparin, none bound actin, 17% bound fibronectin and 67% bound plasminogen. The vast majority of ribosomal proteins (92%) interacted with both actin and plasminogen but only 23% with heparin.

When analysing proteins that were both surface exposed and processed, we noted that the number of cleavage events within protein groups varied, with the P97 and P102 family adhesins showing a significantly higher number of cleavage events compared to other groups (Fig. 5). To assign putative functional data to the cleaved surface proteins, affinity chromatography using heparin, actin, fibronectin and plasminogen as bait was used to enrich *M*. *hyopneumoniae* protein lysates in an untargeted, systems-wide manner. These data were able to ascribe one or more putative binding interactions to 70 of the 75 cleaved surface proteins shown in Table 3. *M*. *hyopneumoniae* proteins interacted with columns coupled with plasminogen and heparin more often than columns combined with actin and fibronectin (Table 3 and Fig. 6). Most of the adhesins and lipoproteins (>88%) were retained in all of the affinity experiments, however the other surface proteins interacted with fewer bait molecules. For example, no proteases or redox proteins were retained on columns coupled with actin and only a small number were recovered from columns coupled with fibronectin. No translation or transcription proteins were retained on columns coupled with fibronectin. Many ribosomal proteins were recovered from affinity columns coupled with actin and plasminogen, but few interacted with columns coupled with heparin (Fig. 6).

We also noted a possible link between the number of cleavage events and binding capabilities. The average number of cleavage events in the ORFs detected in our surfaceome was 5.4. However, those proteolytic products of ORFs that appeared to interact with all four bait molecules in chromatography experiments had an average of 11 cleavage events, whereas those that did not bind any bait molecules had an average of 2.5 (Table 3). Interestingly, while half of the proteins that bound heparin, actin, fibronectin, and plasminogen were adhesins and lipoproteins, the remaining 50% of universal binders (L-lactate dehydrogenase, enolase, pyruvate dehydrogenase, GAPDH, elongation factor Tu, chaperone protein DnaK, and NADH oxidase) have been previously identified as moonlighting proteins in a wide range of organisms^43^. The average number of cleavage events for adhesins was 21, while the average was 13 for the putative moonlighting proteins.

### Proteolytic processing and putative moonlighting functions of glyceraldehyde 3-phosphate dehydrogenase (MHJ_0031)

**Figure 7:**
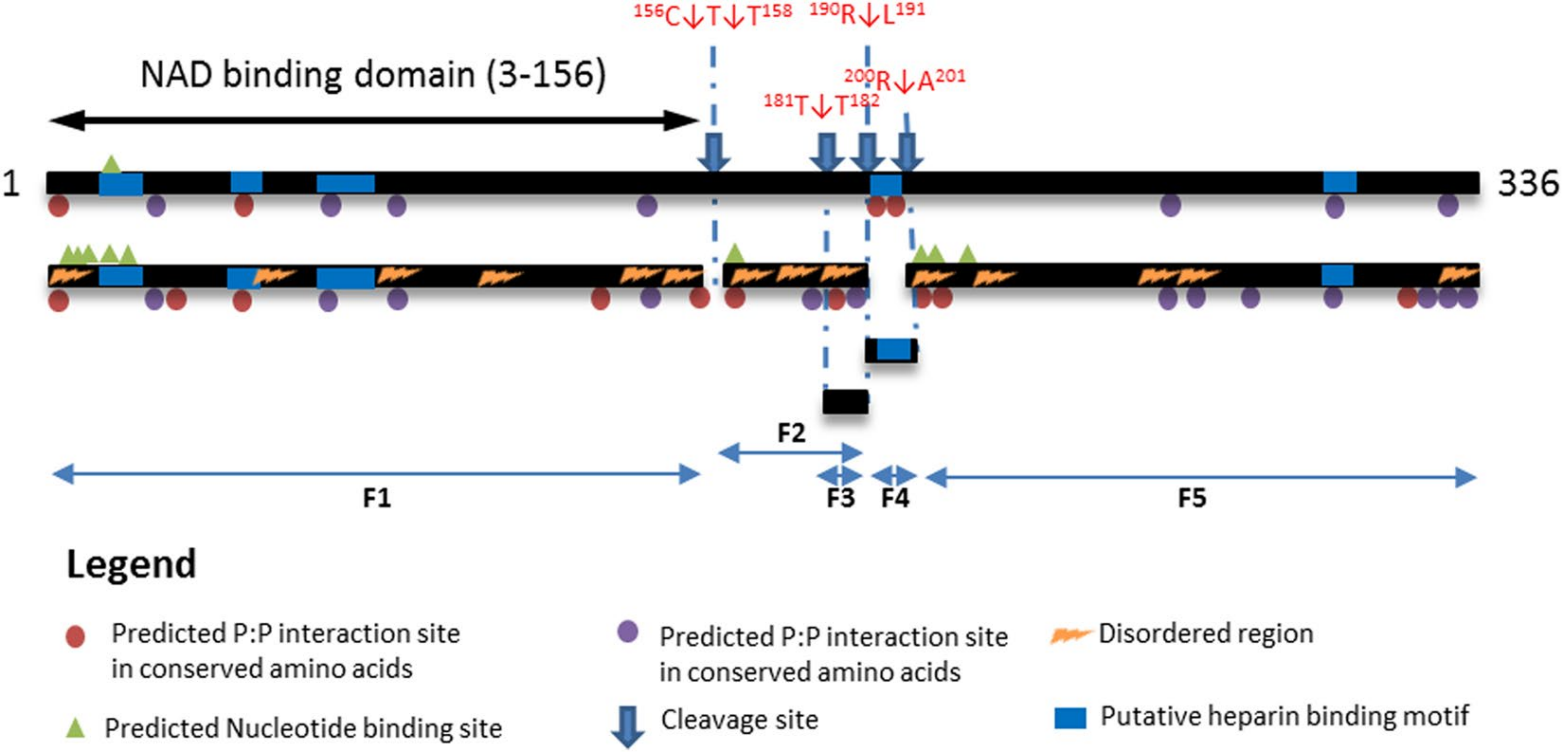
Bioinformatic analysis of full-length *M*. *hyopneumoniae* GAPDH and fragments. The top black line represents full-length *M*. *hyopneumoniae* GAPDH, and black lines beneath it represent fragments generated by cleavages (blue downward arrows) detected by dimethyl labelled peptides. Colored circles represent predicted P:P interaction sites (red circles for sites within conserved amino acids, blue circles for sites within unconserved amino acids). Green triangles represent predicted nucleotide binding sites. Blue boxes represent putative binding regions, and yellow zigzags represent disordered regions. Bioinformatic predictions show that the fragments become disordered post-cleavage and have additional interaction sites that are concentrated at the newly formed termini. F1 constitutes the NAD-binding domain.

Five dimethyl-labelled peptides mapped to glyceraldehyde 3-phosphate dehydrogenase (GAPDH) (Table 5), a protein known to moonlight on the surface of other bacteria^43^. The data indicates that the protein is cleaved into five fragments (F1–F5) (Fig. 7). Our affinity chromatography binding data suggests that *M*. *hyopneumoniae* GAPDH binds actin, fibronectin, heparin and plasminogen (Table 3). Bioinformatic tools were used to compare full-length GAPDH to fragments 1–5 of GAPDH (F1–F5), with respect to predicted disordered regions, nucleotide binding and protein:protein (P:P) interaction sites, putative heparin binding motifs, conserved domains, predicted solvent accessibility, and the evolutionary conversation of amino acids between homologous species.

**Table 5:** Dimethyl-labelled peptides identified in *M*. *hyopneumoniae* GAPDH.

Fragment	Peptide Sequence	E-value
F2	^156^C↓^157^TTNALAPLVNALDKEFGINHGFMTTIHAYTADQR^190^↓L^191^	1.1E^−2^
	^158^T↓^159^TNALAPLVNALDKEFGINHGFMTTIHAYTADQR^190^↓L^191^	3.0E^−4^
F3	^180^T↓^181^TIHAYTADQR^190^↓L^191^	4.8E^−3^
F4	^189^Q↓^190^RLQDAPHGDLR^200^↓R^201^	0.01
	^190^R↓^191^ LQDAPHGDLRR^201^↓A^202^	5.4E^−4^

GAPDH had 11 predicted P:P interaction sites (4 within conserved amino acid regions, seven within unconserved amino acid regions) (Supplementary Data S9), 1 predicted nucleotide binding site and no disordered regions. F1 had four additional putative nucleotide binding sites and three additional putative P:P interaction sites, and six disordered regions. Interestingly, the first cleavage at ^156^C↓T^157^ releases the NAD binding domain from the protein. F2 represents an area devoid of any interaction sites in the parent protein, yet this fragment gained 4 putative P:P interaction sites and one putative nucleotide binding site as well as three disordered regions. F5 gained three new putative nucleotide binding sites and seven additional putative P:P interaction sites. F3 and F4 were too small to undergo bioinformatic analysis. In both GAPDH and fragments of GAPDH, at least 1 P:P interaction site was predicted within a putative heparin-binding region (Fig. 7). In summary, cleaved fragments showed more disorder and additional macromolecule- and nucleotide-binding sites, the majority of which were concentrated at the N and C termini.

## Discussion

The most striking observation of this study was the extent of endoproteolysis that impacted the mature proteome of *M*. *hyopneumoniae*. The 669 labelled neo-N-termini listed in Supplementary Data S7 describe endoproteolytic processing events, often at multiple sites within the same ORF. These N-termini are likely the products of proteolytic cleavages produced by endoproteases of *M*. *hyopneumoniae* origin. Endoproteolysis has been extensively reported in the adhesins of *M*. *hyopneumoniae* using protein-centric methods^35^. However, the N-terminal data presented here demonstrates that endoproteolysis is not restricted to molecules that execute their primary function(s) on the cell surface, but also encompasses proteins that moonlight at cell surface sites. This study represents the first systems-wide analysis of proteolytic processing in a eubacterial organism and is a significant contribution towards understanding the proteolytic landscape of a reduced-genome pathogen.

Previous N-terminome studies in bacteria have identified neo-N-termini among a vast catalogue of tryptic peptides generated during shotgun proteome analyses and a variety of N-terminal enrichment strategies^3^. Here we used dimethyl labelling of genuine neo-N-termini before trypsin digestion, and recovered the N-terminal peptides using charge reversal of internal peptides and strong cation exchange^47^. This method enabled us to recover true N-terminal sequences, with or without iMet processing, for 115 of the 679 ORFs coded by *M*. *hyopneumoniae* strain J. However, we cannot make any assessments whether the N-termini were naturally dimethyl modified using this data. In addition, while we looked for acetylated N-termini, we found no significant hits, thus we excluded this modification from our final search parameters.

Notably, we identified two N-terminal peptides, one with methionine intact and the other where iMet was removed for a subset of *M*. *hyopneumoniae* proteins including MHJ_0125 (a glutamyl aminopeptidase), MHJ_0080 (purine-nucleoside phosphorylase) and MHJ_0506 (phosphate acetyltransferase). In each case, serine or threonine were identified in the P1′ position (Table 2). These observations demonstrate that iMet removal by MAP when S and T reside in the P1′ position may not be as efficient as previously described^4, 45^ or that *M*. *hyopneumoniae* NME is different to that of other bacteria. It was notable that iMet was removed from several proteins when the P1′ position was occupied by large charged residues. Our studies showed that rMHJ_0169 (MAP) was not active against synthetic peptides displaying charged peptides in position P1′, only demonstrating cleavage when alanine was in the P1′ position. This enzymatic behaviour is typical of other well characterised MAPs^48^. The same peptides were then tested against rGAP and rLAP, two proteins previously characterised as surface exposed aminopeptidases in *M*. *hyopneumoniae* ^37, 40^. Interestingly, both rLAP and rGAP cleaved peptides with a basic residue in P1′ but only rLAP cleaved if P2′ was also basic. Additionally, the best substrate for rGAP was MKIISI which was a poor substrate for rLAP (6% cleavage efficiency). These data illustrate the importance of the residue in P2′. While all three aminopeptidases cleaved when alanine was in P1′, rMAP was the most efficient. Collectively, our protease experiments suggest that MAP, LAP and GAP work in concert to perform NME during protein maturation in *M*. *hyopneumoniae*. Furthermore, these aminopeptidases may also play an important role in processing N-terminal sequences of neo N-termini created by an endoproteolytic processing events. In support of this hypothesis, we report the identification of a series of tryptic peptides that differed from one another only by the sequential loss of one or more N-terminal amino acids^33, 35^. Interestingly, the extent of NME on the proteome of *M*. *hyopneumoniae* appears to be limited compared to other organisms^7^. This striking observation may be explained in part by the discovery that many mycoplasmas, including *M*. *hyopneumoniae*, do not possess peptide deformylase (PDF), an enzyme considered integral to protein maturation and NME in bacteria^9^.

During infection, virulence-associated proteins, particularly adhesins, invasins and immunomodulatory proteins, are presented on the bacterial cell surface. These molecules are essential for colonising and invading target host cells and commandeering host cell machinery. Many of these bacterial proteins have the capacity to interact with ECM proteins and components of the host immune system. The surface proteome of *M*. *hyopneumoniae* strain 7448 has previously been catalogued using a biotin labelling protocol. The study identified 59 protein species to be present at the cell surface^49^. Here, we have performed two complementary, orthogonal approaches of cell surface analysis for *M*. *hyopneumoniae* (strain J) to improve coverage and confidence in cell surface protein identifications. In cell shaving experiments, tryptic peptides released by digesting the surface of intact *M*. *hyopneumoniae* cells with trypsin were identified by LC-MS/MS. In separate experiments, proteins on the surface of *M*. *hyopneumoniae* were labeled using NHS-biotin, purified and enriched using avidin chromatography, separated by size using SDS-PAGE and identified by LC-MS/MS. By combining these approaches, we constructed a comprehensive database of proteins and their processed proteoforms that decorate the surface of *M*. *hyopneumoniae*. Of the 671 predicted J strain proteins, 159 were identified on the extracellular surface of *M*. *hyopneumoniae* (strain J), including 47 homologues of surface proteins identified in the study of *M*. *hyopneumoniae* (strain 7448)^49^. The shared proteins were largely made up of the adhesin proteins, membrane proteins and lipoproteins, but also cytosolic moonlighting proteins. 16 proteins identified in the 7448 surfaceome were not identified in the J strain surfaceome analysis, however half of these homologues were also not identified by the exhaustive proteogenome analysis of *M*. *hyopneumoniae* strain J^50^, suggesting a difference in protein abundance between the two strains. Our surfaceome dataset shows that ~24% of the 671 predicted proteins of the *M*. *hyopneumoniae* strain J proteome may be surface accessible. These data include adhesins, lipoproteins, ABC transporters and other proteins with canonical signal secretion peptides in their N-termini. Notably, we also identified many metabolic enzymes and ribosomal proteins on the surface of *M*. *hyopneumoniae* strain J. Proteins that were predicted to be secreted onto the cell surface by SecretomeP only constituted 70/159 (~40%) proteins in our surfaceome, 54 by non-classical secretion mechanisms. Moreover, 70/159 (~40%) of the surface proteome comprised proteins that have a canonical function in the cytosol (putative moonlighting proteins). These protein identifications do not appear to arise from simple cell lysis during experimentation, as a specific profile of ribosomal and metabolic proteins is presented which differs considerably from the complement found in cellular proteome previously catalogued^50^. We have demonstrated that thorough washing of intact cells in PBS before protein shaving or biotinylation had no effect on cell viability based on colony counts (Supplementary Data S10), and is much gentler method than other techniques employed in the field. Previous work also demonstrated that *M*. *hyopneumoniae* cytosolic moonlighting proteins are localised on the cell surface using orthogonal methods including immunofluorescence microscopy^37, 40^. Importantly, many of these metabolic and ribosomal proteins have been identified as surface proteins in other bacteria, with important moonlighting functions during pathogenesis^43, 51, 52^. We speculate that proteolytic processing might be a mechanism to generate novel protein function from these moonlighting proteins after a proteoform traffics to a different cell location, as has been described in many eukaryote systems^2, 53^. This is consistent with the observation that many of the metabolic proteins are capable of interaction with the host molecule heparin (Fig. 5), a highly sulphated glycosaminoglycan present in the porcine lung epithelium, which is a major adhesive target during *M*. *hyopneumoniae* colonisation^54^. Similarly ribosomal proteins demonstrated an ability to bind to host actin, a crucial component of the ECM (Fig. 5). Both classes of proteins show high levels of interaction with host plasminogen (Fig. 5), a key molecule recruited to the cell surface of pathogens during the infection and invasion of host tissues^41^.

Bacteria are known to utilise proteolysis to adapt to changing conditions and manipulate host defences^5^. Surface proteins are cleaved to aid processes including growth, biofilm formation, pathogenesis, stress, and peptide secretion^55^. Regulated proteolysis may reduce a protein into its amino acid constituents to be reused in protein biosynthesis, a process vital for genome-reduced pathogens that are unable to synthesise amino acids, or cleave specifically to produce novel proteoforms^56^. Our previous protein-centric experiments identified 35 proteolytically processed *M*. *hyopneumoniae* proteins, including adhesins, lipoproteins, and moonlighting proteins^35^. However, this list was not exhaustive as many proteins lacked sufficient sequence coverage to be confirmed as cleavage fragments. The N-terminome study described here identified 82 proteolytically cleaved proteins, 75 of which were also identified in our surfaceome.

Consistent with previous findings, we found proteolytic events occurring at hydrophobic residues in the P1 position in members of the P97 and P102 adhesin families (^761^n-V↓a-V-s^766^ in P97 paralogue; ^192^T-n-F↓a-D^196^ in P123 homologue; and ^343^-T-e-F↓v-E^347^ in the Mhp683 homologue), and many cleavages consistent with trypsin-like activity^32, 33^. A lon protease and an ATP-dependent zinc metalloprotease, FtsH, were identified in our surfaceome. Both proteases share a preference for hydrophobic residues at the P1 position. Additionally, endopeptidase activity when phenylalanine resides in the P1 position has been described and it was suggested that a subtilisin-like protease might be responsible for performing these processing events^57^. Lon and FtsH proteases may be responsible for the preponderance of proteolytic events targeting hydrophobic residues, but this remains to be validated experimentally. While FtsH has some propensity for cleaving basic residues, the sheer number of cleavages at arginine (69% of total cleavage events in our N-terminome) suggests that an unidentified trypsin-like protease is produced on the surface of *M*. *hyopneumoniae*.

The genome of *M*. *hyopneumoniae* is predicted to encode 270 putative uncharacterised proteins. *In silico* analyses of these uncharacterised proteins identified two putative subtilisin-like proteases (MHJ_0398 and MHJ_0332), a second putative glutamyl aminopeptidase (MHJ_0496), and a protein (MHJ_0568) predicted to be a secreted protease with a trypsin-like domain. MHJ_0568 may be responsible for the trypsin-like cleavage events that were frequently observed in *M*. *hyopneumoniae* surface proteins. Approximately half of the surface proteins we identified were processed by proteolysis, many in a trypsin-like manner. Such extensive processing is akin to ectodomain shedding, in which proteases known as sheddases, release membrane-bound proteins. Sheddases regulate cell adhesion, migration, proliferation, and cell-to-cell communication^58^. While a recent study found a vast number of peptides derived from an extensive repertoire of proteins in the secretome of culture supernatants of *Lactococcus lactis* ^59^, ectodomain shedding currently remains a largely eukaryotic phenomenon. Sheddases are typically matrix metalloproteinases (MMP) or members of a transmembrane protease family called ADAMs^60^. *M*. *hyopneumoniae* is not predicted to encode for either an ADAM or MMP, however trypsin-like sheddases have been described previously^32, 33^. Our data reconfirms and extends previous findings that ectodomain shedding may play a vital role in cellular behaviour and pathogenesis in Mycoplasma spp.^32, 35, 61^.

Interestingly, three trypsin-like proteases are currently candidates for the generation of the A-signal, a quorum-sensing-like system used by *Myxococcus xanthus*. In contrast to using secreted autoinducers, the A-signal utilises peptides as signalling molecules. Individual amino acids have A-signal activity, and the activity of a cleaved fragment is equal to the sum of the A-signal activity of its amino acids^62^. *M*. *xanthus* uses the A-signal to initiate aggregation of cells to ultimately differentiate into spores. It has been suggested that secreted trypsin-like proteases non-specifically degrade surface-exposed proteins to generate the A-signal during early starvation. While *M*. *hyopneumoniae* is not known to form spores, a similar intercellular communication system could be utilised to initiate biofilm formation. Work in our laboratory has shown that *M*. *hyopnumoniae* forms biofilms on abiotic surfaces, on cell monolayers and in the respiratory tract of swine (Raymond and Djordjevic, unpublished data).

Proteolytic processing on the cell surface of *M*. *hyopneumoniae* may also release peptides from the cell surface into the external environment, where they may function as intracellular signals, competitive inhibitors or immunogenic decoys. A recent study of the secretome of *M*. *hyopneumoniae* revealed that 62 mycoplasmal proteins are released into the media during culture *in vitro* ^63^, although it is not known whether these proteins exist as intact molecules or processed fragments. Interestingly the array of secreted proteins found by that study included many classical “cytosolic” proteins with no known mechanism of secretion and the list of proteins showed considerable overlap with the proteins identified in our surfaceome. In our study, proteolytic fragments were retained on the cell surface albeit by an unknown mechanism. Protein fragments may benefit a genome-reduced pathogen because they show more protein disorder compared to the parent proteoform^64^. Intrinsically disordered proteins and peptides present more protein:protein (P:P) interaction sites, bind multiple partners, provide resistance to non-native conditions, and are targets of post-translational modification events (PTMs)^65, 66^.

GAPDH is considered the quintessential example of a moonlighting protein with functional roles in cell adhesion, membrane trafficking and regulation of gene expression^67^. We found *M*. *hyopneumoniae* GAPDH to be surface exposed and proteolytically cleaved into five fragments. GAPDH was predicted to have 11 putative P:P interaction sites, one putative nucleotide binding site, and no disordered regions. Our bioinformatic analysis demonstrated that cleavage fragments of glyceraldehyde 3-phosphate dehydrogenase (GAPDH) gained 14 disordered regions, 12 additional putative P:P sites and eight additional putative nucleotide binding sites (Fig. 7). Importantly, most of the additional interaction sites were predominantly localised to newly formed N- and C-termini, regions where most ascribed functions to disordered regions lie^68^. Equipped with the knowledge that disordered regions are concentrated at protein tails and facilitate PTMs, it was interesting to note that our cleavage data identified the most common amino acids at P1′ position and therefore the first amino acids of newly formed N-termini were lysine, serine, and threonine. Protein acetylation is a PTM that often occurs at lysine residues where it influences protein stability and immunogenicity^69^. Protein phosphorylation commonly occurs at serine and threonine residues and is known to regulate bacterial cell signaling pathways, lipid metabolism, protein interactions, and avoidance of antibiotics^69^. By generating and retaining protein fragments that are more disordered and receptive of PTMs, *M*. *hyopneumoniae* can potentially expand protein function. This hypothesis is supported by a distinct correlation between protein cleavage frequency and putative binding capacity in affinity chromatography experiments. The average number of cleavage events in all surface exposed proteins was 5. Those proteins that were retained in all the affinity columns (four different bait molecules) had an average of 11 cleavage events. Proteins not retained during by affinity chromatography had an average of 3 cleavage events. The adhesins were the most extensively processed proteins, are multifunctional and are targets the greatest numbers of cleavage events (an average of 21). This data suggests proteolytic processing results in fragments which gain binding capabilities.

In conclusion, this study is the first to examine endoproteolytic processing in prokaryotes using a systems-wide approach. Many of the observations derived from the characterisation of chemically labelled N-termini both confirm and expand previous findings that describe proteolytic processing events^28^ and verify ORF predictions from an earlier proteogenomic study of *M*. *hyopneumoniae* ^50^. We characterised the function of MAP and extended the knowledge of two aminopeptidases with regard to how they contribute to NME. We expand upon the known surfaceome of *M*. *hyopneumoniae* ^49^ and describe novel processing events in surface-exposed proteins. Our affinity chromatography studies suggest that cleavage fragments generated from a wide variety of surface molecules can interact with functionally and structurally-diverse host molecules. Our data is consistent with the notion that protein moonlighting is a widespread phenomenon in prokaryotes. Proteolytic processing represents a novel mechanism to expand protein function and generates new opportunities to develop therapies to treat this economically and environmentally devastating pathogen.

## Methods

### Mycoplasma hyopneumoniae culture conditions

*M*. *hyopneumoniae* cells were grown in modified Friis media^70^ for 48 h at 37 °C with continuous agitation. Cells were pelleted by centrifugation at 10,000 × g for 10 min, re-suspended with Phosphate Buffered Saline (PBS) [Sigma-Aldritch] and centrifuged for 10 minutes at 10,000 × g, repeated 3 times to remove serum and secreted proteins.

### Dimethyl labelling and enrichment of *M*. *hyopneumoniae* proteins

*M*. *hyopneumoniae* cells were resuspended in 6 M guanidine hydrochloride with Complete protease inhibitors [Roche] and lysed with an ultrasonic probe at 50% power (performed in triplicate). Lysates were reduced using 5 mM tributylphosphine [Sigma-Aldritch] and alkylated 20 mM acrylamide monomers [Sigma-Aldritch] for 90 minutes at room temperature. Proteins were precipitated in 10 volumes of acetone for 3 hours at −80 °C, pelleted by centrifugation at 20,000 × g for 20 minutes and resuspended in 100 mM HEPES solution adjusted to pH 7. Protein labelling was performed on 1 mg of *M*. *hyopneumoniae* (strain J) protein by the addition of 20 mM formaldehyde (ultrapure grade) [Polysciences, USA] and 20 mM sodium cyanoborohydride [Sigma-Aldritch], every 30 minutes to a final concentration of 60 mM in a final volume of 1 mL and incubated at 37 °C for a minimum 4 hours^47^. The reaction was quenched by the addition of 100 mM Tris for 30 minutes at 37 °C and then precipitated with 8 volumes of acetone and 1 volume of methanol at −80 °C for 3 hours. The precipitated protein was then pelleted by centrifugation at 20,000 × g for 20 minutes and washed with 5 volumes of methanol. The protein pellet was resuspended in 50 mM sodium hydroxide, pH 8.0 and digested with Trypsin Gold [Promega, USA] overnight at 37 °C. Digested peptides were then labelled with 60 mM formyl-sulphobenzene [Sigma-Aldritch] and 60 mM sodium cyanoborohydride [Sigma-Aldritch] at 37 °C for a minimum 4 hours, as previously described^47^. The sample was diluted using Strong Cation Exchange buffer A (10% acetonitrile, 20 mM ammonium dihydrogen phosphate, pH 2.7) and injected onto a 100 × 2.1mm PolySULFOETHYL A column with 5um 200 Å particles [PolyLC, USA], using a Shimadzu Nextera × 2 UHPLC at 0.2 mL.min^−1^. Sulphonated internal and C-terminal peptides were collected from the flowthrough fraction. Dimethylated N-terminal peptides were then eluted with an increasing concentration of NaCl to 1 M over 30 minutes. The acetonitrile was evaporated using a speedvac, desalted by SiliaPrepX™ HLB Polymeric SPE cartridges [Silicycle, Canada], and analysed by LC-MS/MS.

### Liquid chromatography tandem mass spectrometry (LC-MS/MS)

Using an Eksigent 415 autosampler connected to a 415 nanoLC system (Eksigent, USA), 5 µL of the sample was loaded at 300 nl/min with MS buffer A (2% Acetonitrile, 0.2% Formic Acid) by direct injection onto a PicoFrit column (75 µmID × 150 mm; New Objective, Woburn, MA) packed with C18AQ resin (1.9 µm, 200 Å; Dr Maisch, Germany). Peptides were eluted from the column and into the source of a 6600 TripleTOF hybrid quadrupole-time-of-flight mass spectrometer (Sciex, CA) using the following program: 2–35% MS buffer B (80% Acetonitrile, 0.2% Formic Acid) over 90 minutes, 35–95% MS buffer B over 9 minutes, 95% MS buffer B for 9 minutes, 80–2% for 2 min. The eluting peptides were ionised at 2300 V. An Intelligent Data Acquisition (IDA) experiment was performed, with a mass range of 350–1500 Da continuously scanned for peptides of charge state 2 + −5 + with an intensity of more than 400 counts/s. Up to 50 candidate peptide ions per cycle were selected and fragmented and the product ion fragment masses measured over a mass range of 100–2000 Da. The mass of the precursor peptide was then excluded for 15 seconds.

### Mass Spectrometry Data Analysis

The MS/MS data files produced by the 6600 TripleTOF were searched using Mascot Daemon (version 2.4; Perkins, D.N. 1999) against the MSPnr100 database (based on the major reference sequence repositories of NCBI, Refseq, UniProtKB, EupathDB and Ensembl. Duplicate entries at the species-level are removed, “multispecies” entries (NCBI) and “fragments” (TrEMBL) are ignored. vQ115). The search parameters were: Precursor tolerance 10 ppm and product ion tolerances ±0.2 Da; charge states: 2+, 3+ and 4+; Propionamide (C), Dimethyl (K) specified as fixed modifications; oxidation (M), deamidation (NQ), Dimethyl (N-term) specified as variable modifications; enzyme specificity was semi-ArgC; 1 missed cleavage was allowed. The results of the search were then filtered by including only peptides with a dimethyl labelled N-terminus and excluding peptides with a p-value greater than 0.05.

### Expression and purification of *M*. *hyopneumoniae* aminopeptidases

The expression and purification of *M*. *hyopneumoniae* glutamyl aminopeptidase and leucine aminopeptidase have been described previously^37, 40^. Similarly, the *mhj_0169* gene predicted to encode for a methionine aminopeptidase in *M*. *hyopneumoniae* was synthesized and cloned into the expression vector PS100030 by Blue Heron Biotech (USA) converting all in frame TGA codons, which encode for tryptophan in mycoplasmas, to TGG. The recombinant construct was transformed into BL21 (Invitrogen, USA) using standard protocols outlined in the manufacturer’s instructions. Polyhistidine tagged rMHJ_0169 was purified under native conditions using 50% slurry of Profinity immobilized metal affinity chromatography Ni^2+^-charged resin (Bio-Rad) as per the manufacturer’s instructions. The eluted protein was dialysed against PBS in 10 K MWCO dialysis tubing and stored at −20 °C.

### Aminopeptidase activity assays

To determine MAPs specificities for N-terminal amino acid cleavage, 30 nM rMHJ_0169 was added to 50 µM AMC-coupled substrates (A; R; E; I; V; G; L; F; P; M-AMC) in combination with 1 mM metal cofactors (Zn^2+^, Ca^2+^, Cu^2+^, Mn^2+^, Mg^2+^ and Co^2+^) and a range of pH conditions (50 mM of either sodium acetate (pH 4–5.5), Tris-HCl (pH 6–8.8) or sodium borate (pH 10). Once best cofactor was ascertained, activity was optimised using a concentration gradient from 1 µM to 1 mM. Fluorescence was measured using a 96-well ELISA plate with a Synergy HT multi-mode microplate reader (BioTek, USA) linked to gen5 v. 1.08 software (BioTek). Reactions were mixed for 2 s immediately prior to fluorometric analysis. Assays were read every 60 s for 1 h at a wavelength of 360 nm and 460/40 nm at 37 °C.

To determine aminopeptidases responsible for NME in *M*. *hyopneumoniae*, 5 peptides were synthesised by Chempeptide Limited (China) (Met-Lys-Lys-Ile-Ala-Ile; Met-Asp-Lys-Lys-Leu-Glu; Met-Ala-Lys-Glu-Ile-Ile; Met-Lys-Ile-Ile-Ser-Ile; Met-Lys-Asn-Ile-Val-Asp) and solubilized in water to a concentration of 1 mg/ml. For each peptide, 1 µl was diluted in 8.5 µL 50 mM Tris-HCl buffer (pH 7.5) and 0.5 µL 100 mM Co^2+^. Purified rMHJ_0169, rMHJ_0659, or rMHJ_0125 was added in a 1:20 protease to substrate concentration and incubated for 60 min at 37 °C. The peptides were then desalted and captured using C18 ZipTips (Millipore, USA). 1 µl of peptide sample was then spotted onto a clean 384-well OptiTOF target plate (AB Sciex, USA) followed by 1 µl of 5 mg/ml α-Cyano-4-hydroxycinnamic acid (CHCA) dissolved in 50% acetonitrile, 0.1% triflouroacetic acid, 10 mM ammonium dihydrogen phosphate and allowed to dry. Spotted samples were then analysed using a Sciex 5800 MALDI-TOF/TOF MS in positive reflector mode. Laser intensity was set to 2600 for MS parent ion scans and 3000 for MS/MS fragmentation ion scans. 400 laser shots were averaged for MS scans and up to 1250 shots were averaged for MS/MS scan with the Dynamic Exit algorithm selected, which monitors spectral quality and stops shot accumulation if a user defined threshold is met. MS parent ion scans were calibrated using the TOF/TOF standards mixture (Sciex) whereas MS/MS scans were calibrated with fragments of Glu-Fibrinopeptide B. The resulting MS spectral data was then manually inspected to explain the ions present with reference to their amino acid sequence and the cleavage events caused by N-terminal proteolysis.

### *M*. *hyopneumoniae* enzymatic cell surface shaving

Freshly harvested *M*. *hyopneumoniae* cells were washed extensively (>3 times) in PBS and pelleted by centrifugation (4,000 × g 10 mins 4 °C). Cells were resuspended in PBS (pH 7.8) and enzymatic cell shaving using trypsin was performed at 37 °C for 5 min. Intact cells were pelleted by centrifugation and the supernatant containing liberated surface proteins collected on ice to cease trypsin activity. Surface proteins were analysed by 1D gel electrophoresis or further digested to peptides with trypsin prior to analysis by SCX and LC-MS/MS as described previously^38^.

### Cell surface biotinylation of *M*. *hyopneumoniae* proteins

Freshly harvested *M*. *hyopneumoniae* cells were washed extensively (>3 times) in PBS and pelleted by centrifugation (4,000 × g 10 mins 4 °C). Cells were resuspended in PBS (pH 7.8) and biotinylated with Sulfo-NHS-LC Biotin [Thermo Scientific] for 30 seconds on ice. The reaction was then quenched with the addition of a final concentration of 50 mM Tris-HCl (pH 7.4) and incubated for 15 mins. Cells were washed in three changes of PBS and pelleted by centrifugation. Cells were lysed and precipitated protein was pelleted, air dried and resuspended in buffers appropriate for downstream application as previously described^29, 38, 39^. Approximately 1 mg of protein was subjected to avidin affinity chromatography in a column packed with 1 ml of immobilized monomeric avidin [Thermo Scientific], separated by 1D-SDS-PAGE into 16 size fractions and analysed on a QSTAR Elite on 60 minute gradients as described previously^25^.

### Protein enrichment using column affinity chromatography

Immobilised heparin affinity chromatography, avidin purification of actin-, plasminogen- and fibronectin interacting proteins were loaded into a column. *M*. *hyopneumoniae* protein lysates were loaded onto the column using PBS (pH 7.8) and non-binding proteins washed with 4 × column volumes (5 mL) of PBS. Binding proteins were eluted with 2 M NaCl (heparin); 30% acetonitrile, 0.4% trifluoroacetic acid (actin and plasminogen); or 7 M urea, 2 M thiourea, 40 mM Tris and 1% (w/v) C7BzO [Sigma-Aldrich] (fibronectin). Protein elutions were separated by 1D-SDS-PAGE and fractions were analysed on a QSTAR Elite on 60 minute gradients as described previously^32–34^. Negative controls were performed with avidin agarose without bait host molecules. These controls demonstrated that *M*. *hyopneumoniae* lysate proteins were unable to bind to the columns as they were not found in the elutions after washing (Supplementary Data S11).

### Bioinformatic Analysis

The putative functions of *M*. *hyopneumoniae* were assigned by the Uniprot database and predicted cell localisations were determined by PSORTb^71^ and SecretomeP^72^. The bioinformatic tools used to compare *M*. *hyopneumoniae* GAPDH to its fragments generated post endoproteolytic cleavage include Meta-Disorder^73^ to predict disordered regions, ISIS to predict P:P and nucleotide binding sites^74^, ScanProsite^75^ to find putative heparin binding motifs (using search parameters X-B-X(0,2)-B-X(0,2)-B-X and X-B-X(1,4)-B-X(1,4)-B-X, where B = basic residue), NCBI Sequence Viewer^76^ and Conserved Domains Database^77^ to view conserved domains, and ConSurf^78^ to identify evolutionary conversation of amino acids and predicted solvent accessibility. Sequence logos were generated by Seq. 2Logo^79^.

### Availability of Materials and Data

The datasets generated and analysed during this study are available from the corresponding author on reasonable request.

## Electronic supplementary material

Supplementary Data 1

Supplementary Data 2
