# Supplementary material for: N-terminomics identifies widespread endoproteolysis and novel methionine excision in a genome-reduced bacterial pathogen
Source: Sci Rep. 2017 Sep 11;7:11063. doi: 10.1038/s41598-017-11296-9 (PMC5593965; doi:10.1038/s41598-017-11296-9)
Supplement: Supplementary file 2 — Supplementary Data 2 [file 41598_2017_11296_MOESM2_ESM.doc]

**N-terminomics identifies widespread endoproteolysis and novel methionine excision in a genome-reduced bacterial pathogen.**

Iain J. Berry+, Veronica M. Jarocki+, Jessica L. Tacchi, Benjamin B. A. Raymond, Michael Widjaja, Matthew P. Padula, Steven P. Djordjevic

**Supplementary Data**

# Supplementary Data S4: BLAST analysis of peptide deformylases (PDFs) in *Mycoplasma spp*.

The Uniprot database identified the *pdf* gene in the following sequenced *Mycoplasma spp.*


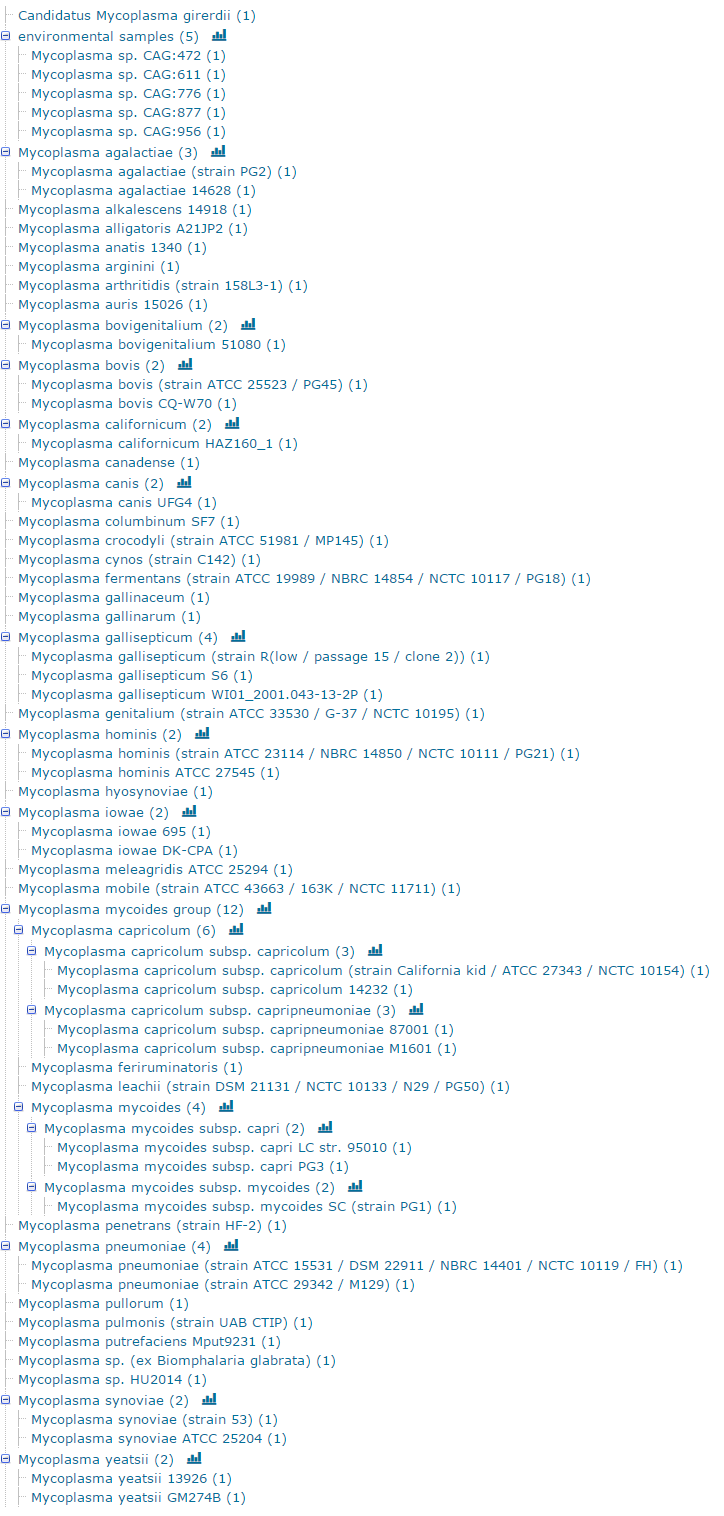


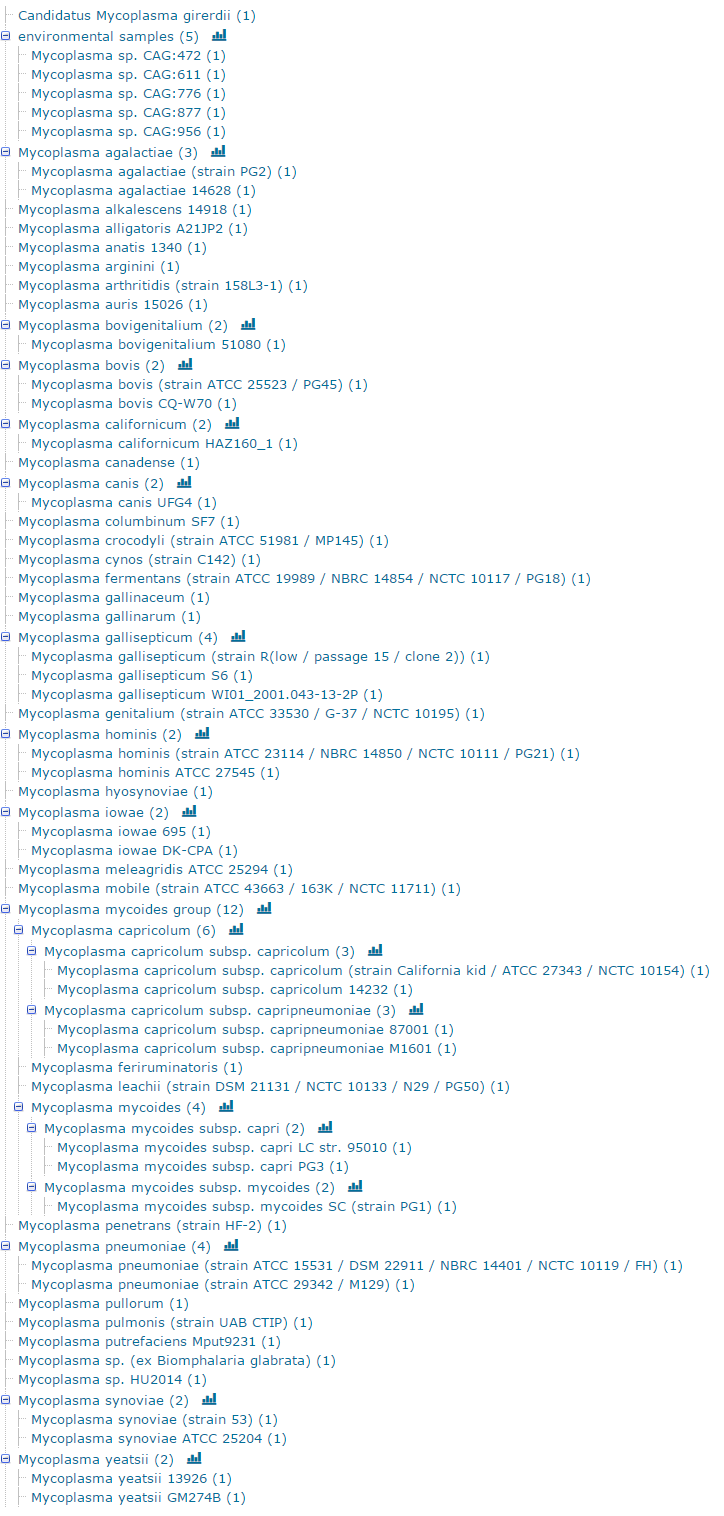


**BLAST searches (tab file format) for PDF in *Mycoplasma hyosynoviae* showing no *M. hyopneumoniae* matches.**

| **Entry** | **Info** |  |  | **Status** |
| --- | --- | --- | --- | --- |
| A0A063YAC7 | E-value: 920E-135; | Score: 981; | Ident.: 100.0% | unreviewed |
| N9UAD5 | E-value: 5.2E-90; | Score: 699; | Ident.: 71.2% | unreviewed |
| N9TRB3 | E-value: 10E-90; | Score: 697; | Ident.: 69.6% | unreviewed |
| A0A077L790 | E-value: 6.1E-87; | Score: 679; | Ident.: 66.8% | unreviewed |
| A0A0C6G2F0 | E-value: 260E-87; | Score: 668; | Ident.: 67.4% | unreviewed |
| A0A1L4FSH9 | E-value: 1.5E-78; | Score: 623; | Ident.: 63.1% | unreviewed |
| B3PN62 | E-value: 2.3E-78; | Score: 622; | Ident.: 59.8% | unreviewed |
| A0A097NSQ3 | E-value: 9.4E-78; | Score: 618; | Ident.: 64.1% | unreviewed |
| D1J7P7 | E-value: 9.4E-78; | Score: 618; | Ident.: 64.1% | unreviewed |
| D4XWB8 | E-value: 3.4E-72; | Score: 581; | Ident.: 63.1% | unreviewed |
| F9QDM4 | E-value: 150E-72; | Score: 570; | Ident.: 59.8% | unreviewed |
| A0A168RJL0 | E-value: 630E-72; | Score: 566; | Ident.: 66.1% | unreviewed |
| C4XE56 | E-value: 15E-69; | Score: 558; | Ident.: 61.3% | unreviewed |
| D5E4Q1 | E-value: 160E-69; | Score: 550; | Ident.: 59.1% | unreviewed |
| Q6KH28 | E-value: 22E-66; | Score: 536; | Ident.: 52.7% | unreviewed |
| A0A0D5ZJP8 | E-value: 34E-66; | Score: 535; | Ident.: 60.2% | unreviewed |
| I1A7I5 | E-value: 34E-66; | Score: 535; | Ident.: 54.4% | unreviewed |
| A0A0F6ZLM2 | E-value: 96E-66; | Score: 532; | Ident.: 55.0% | unreviewed |
| L0RVV9 | E-value: 2.2E-63; | Score: 523; | Ident.: 55.3% | unreviewed |
| E4Q0J2 | E-value: 8.5E-63; | Score: 519; | Ident.: 58.6% | unreviewed |
| A0A059Y7Z0 | E-value: 8.5E-63; | Score: 519; | Ident.: 58.6% | unreviewed |
| D3VPL5 | E-value: 17E-63; | Score: 517; | Ident.: 57.1% | unreviewed |
| A5IXW9 | E-value: 25E-63; | Score: 516; | Ident.: 56.6% | unreviewed |
| I5D5M9 | E-value: 70E-63; | Score: 513; | Ident.: 56.6% | unreviewed |
| A0A1L7MYC6 | E-value: 180E-63; | Score: 511; | Ident.: 51.3% | unreviewed |
| N9TUH4 | E-value: 720E-63; | Score: 507; | Ident.: 50.8% | unreviewed |
| F9UJ31 | E-value: 4.2E-60; | Score: 502; | Ident.: 56.1% | unreviewed |
| A0A059XMQ5 | E-value: 2.1E-57; | Score: 484; | Ident.: 49.0% | unreviewed |
| A0A060PYH4 | E-value: 2.1E-57; | Score: 484; | Ident.: 49.0% | unreviewed |
| A0A0F5H121 | E-value: 4.0E-57; | Score: 482; | Ident.: 55.4% | unreviewed |
| Q4A730 | E-value: 39E-57; | Score: 475; | Ident.: 51.4% | unreviewed |
| A0A0E3H7N1 | E-value: 55E-57; | Score: 474; | Ident.: 51.4% | unreviewed |
| Q98PN3 | E-value: 810E-54; | Score: 447; | Ident.: 48.6% | reviewed |
| A0A0E9DCV7 | E-value: 6.2E-45; | Score: 394; | Ident.: 66.4% | unreviewed |
| A0A1S2QKD7 | E-value: 1.2E-39; | Score: 365; | Ident.: 45.7% | unreviewed |
| A0A0M1NRY7 | E-value: 2.4E-39; | Score: 363; | Ident.: 45.9% | unreviewed |
| A0A0Q9VJA7 | E-value: 2.4E-39; | Score: 363; | Ident.: 45.9% | unreviewed |
| A0A1N7G9P6 | E-value: 2.4E-39; | Score: 363; | Ident.: 47.5% | unreviewed |
| A0A0M1NH25 | E-value: 2.4E-39; | Score: 363; | Ident.: 45.9% | unreviewed |
| A0A090YP43 | E-value: 2.7E-39; | Score: 362; | Ident.: 44.8% | unreviewed |
| A0A1Q9YCV0 | E-value: 5.6E-39; | Score: 360; | Ident.: 45.2% | unreviewed |
| A0A127D2P4 | E-value: 9.5E-39; | Score: 359; | Ident.: 45.9% | unreviewed |
| K0IZ53 | E-value: 12E-39; | Score: 358; | Ident.: 44.2% | unreviewed |
| A0A1J9TRH6 | E-value: 15E-39; | Score: 357; | Ident.: 45.8% | unreviewed |
| A0A098ETC0 | E-value: 19E-39; | Score: 357; | Ident.: 45.3% | unreviewed |
| A0A150F0Y8 | E-value: 21E-39; | Score: 356; | Ident.: 45.2% | unreviewed |
| A0A151UUA7 | E-value: 21E-39; | Score: 356; | Ident.: 45.2% | unreviewed |
| H1BK35 | E-value: 22E-39; | Score: 356; | Ident.: 44.1% | unreviewed |
| U5F950 | E-value: 22E-39; | Score: 356; | Ident.: 44.1% | unreviewed |
| A0A1C5XL96 | E-value: 22E-39; | Score: 356; | Ident.: 44.1% | unreviewed |
| A0A0Q9VJ23 | E-value: 27E-39; | Score: 356; | Ident.: 45.3% | unreviewed |
| A0A0Q6HYP4 | E-value: 27E-39; | Score: 356; | Ident.: 45.3% | unreviewed |
| A8R7Q1 | E-value: 31E-39; | Score: 355; | Ident.: 42.9% | unreviewed |
| B7HMU5 | E-value: 43E-39; | Score: 354; | Ident.: 45.2% | unreviewed |
| C2MPV8 | E-value: 43E-39; | Score: 354; | Ident.: 45.2% | unreviewed |
| F0PST4 | E-value: 43E-39; | Score: 354; | Ident.: 45.2% | unreviewed |
| A0A0S3C191 | E-value: 43E-39; | Score: 354; | Ident.: 45.8% | unreviewed |
| A0A136F2I2 | E-value: 43E-39; | Score: 354; | Ident.: 45.2% | unreviewed |
| A0A0G8ELV3 | E-value: 43E-39; | Score: 354; | Ident.: 45.2% | unreviewed |
| J8JIF5 | E-value: 43E-39; | Score: 354; | Ident.: 45.2% | unreviewed |
| J8GBX2 | E-value: 43E-39; | Score: 354; | Ident.: 45.2% | unreviewed |
| A0A1J9YRA3 | E-value: 43E-39; | Score: 354; | Ident.: 45.2% | unreviewed |
| A0A1J9YIC9 | E-value: 43E-39; | Score: 354; | Ident.: 45.2% | unreviewed |
| B9IW54 | E-value: 43E-39; | Score: 354; | Ident.: 45.2% | unreviewed |
| Q731Z2 | E-value: 43E-39; | Score: 354; | Ident.: 45.2% | unreviewed |
| C2S858 | E-value: 43E-39; | Score: 354; | Ident.: 45.2% | unreviewed |
| A0A0T8XAR8 | E-value: 43E-39; | Score: 354; | Ident.: 45.2% | unreviewed |
| C2QXE5 | E-value: 55E-39; | Score: 353; | Ident.: 45.2% | unreviewed |
| A0A1J9T8N5 | E-value: 60E-39; | Score: 353; | Ident.: 45.2% | unreviewed |
| A0A0N7JJX8 | E-value: 60E-39; | Score: 353; | Ident.: 45.2% | unreviewed |
| A0A1G6ST37 | E-value: 60E-39; | Score: 353; | Ident.: 45.2% | unreviewed |
| A0A0J7HCZ4 | E-value: 60E-39; | Score: 353; | Ident.: 45.2% | unreviewed |
| Q4MTG2 | E-value: 60E-39; | Score: 353; | Ident.: 45.2% | unreviewed |
| C2PJD8 | E-value: 60E-39; | Score: 353; | Ident.: 45.2% | unreviewed |
| A0A1C4EKV1 | E-value: 60E-39; | Score: 353; | Ident.: 45.2% | unreviewed |
| A0A0E1MJ98 | E-value: 60E-39; | Score: 353; | Ident.: 45.2% | unreviewed |
| A0A1D3P5Q4 | E-value: 60E-39; | Score: 353; | Ident.: 45.2% | unreviewed |
| A0A1D3NMW9 | E-value: 60E-39; | Score: 353; | Ident.: 45.2% | unreviewed |
| A0A1D3QS18 | E-value: 60E-39; | Score: 353; | Ident.: 44.6% | unreviewed |
| A0A1J9S9Y9 | E-value: 60E-39; | Score: 353; | Ident.: 45.2% | unreviewed |
| A0A1J9S6A5 | E-value: 60E-39; | Score: 353; | Ident.: 45.2% | unreviewed |
| A0A1C0BW12 | E-value: 63E-39; | Score: 353; | Ident.: 47.3% | unreviewed |
| C3GN76 | E-value: 78E-39; | Score: 352; | Ident.: 45.2% | unreviewed |
| C2TKU5 | E-value: 78E-39; | Score: 352; | Ident.: 45.2% | unreviewed |
| C3G7D9 | E-value: 84E-39; | Score: 352; | Ident.: 45.2% | unreviewed |
| Q6HEJ1 | E-value: 84E-39; | Score: 352; | Ident.: 45.2% | unreviewed |
| C3HMY5 | E-value: 84E-39; | Score: 352; | Ident.: 45.2% | unreviewed |
| C2VY39 | E-value: 84E-39; | Score: 352; | Ident.: 45.2% | unreviewed |
| C3F685 | E-value: 84E-39; | Score: 352; | Ident.: 45.2% | unreviewed |
| B3Z6J6 | E-value: 84E-39; | Score: 352; | Ident.: 45.2% | unreviewed |
| A0A0U0E4Y5 | E-value: 84E-39; | Score: 352; | Ident.: 45.2% | unreviewed |
| D8H8V5 | E-value: 84E-39; | Score: 352; | Ident.: 45.2% | unreviewed |
| A0A154B2G8 | E-value: 84E-39; | Score: 352; | Ident.: 45.2% | unreviewed |
| A0A0B6BU06 | E-value: 84E-39; | Score: 352; | Ident.: 45.2% | unreviewed |
| A0A0K6MNI2 | E-value: 84E-39; | Score: 352; | Ident.: 44.6% | unreviewed |
| A0A0E0W5A0 | E-value: 84E-39; | Score: 352; | Ident.: 45.2% | unreviewed |
| B7JKU8 | E-value: 84E-39; | Score: 352; | Ident.: 45.2% | unreviewed |
| A0A0B6C373 | E-value: 84E-39; | Score: 352; | Ident.: 45.2% | unreviewed |
| A0A0G8E9R2 | E-value: 84E-39; | Score: 352; | Ident.: 45.2% | unreviewed |
| Q81MQ9 | E-value: 84E-39; | Score: 352; | Ident.: 45.2% | reviewed |
| A0A0M0EQ13 | E-value: 100E-39; | Score: 352; | Ident.: 44.8% | unreviewed |
| A0A0F5RSX4 | E-value: 120E-39; | Score: 351; | Ident.: 44.6% | unreviewed |
| J8WXJ9 | E-value: 120E-39; | Score: 351; | Ident.: 44.6% | unreviewed |
| A0A1E8A6V8 | E-value: 120E-39; | Score: 351; | Ident.: 44.6% | unreviewed |
| N1LUA1 | E-value: 120E-39; | Score: 351; | Ident.: 44.1% | unreviewed |
| E2SJQ4 | E-value: 120E-39; | Score: 351; | Ident.: 43.7% | unreviewed |
| A0A0G8CJB7 | E-value: 120E-39; | Score: 351; | Ident.: 44.6% | unreviewed |
| A0A1C4FQ85 | E-value: 120E-39; | Score: 351; | Ident.: 44.6% | unreviewed |
| A0A1C4FQC0 | E-value: 120E-39; | Score: 351; | Ident.: 44.6% | unreviewed |
| A0A1J9UDX9 | E-value: 120E-39; | Score: 351; | Ident.: 44.6% | unreviewed |
| J8KWH5 | E-value: 170E-39; | Score: 350; | Ident.: 44.1% | unreviewed |
| J8QTJ8 | E-value: 170E-39; | Score: 350; | Ident.: 44.1% | unreviewed |
| A0RHY6 | E-value: 170E-39; | Score: 350; | Ident.: 45.2% | unreviewed |
| A0A1A9PXX6 | E-value: 170E-39; | Score: 350; | Ident.: 44.6% | unreviewed |
| R8KSE1 | E-value: 170E-39; | Score: 350; | Ident.: 44.6% | unreviewed |
| J8SQU4 | E-value: 170E-39; | Score: 350; | Ident.: 44.1% | unreviewed |
| A0A0K6J489 | E-value: 170E-39; | Score: 350; | Ident.: 44.6% | unreviewed |
| B3ZRP7 | E-value: 170E-39; | Score: 350; | Ident.: 45.2% | unreviewed |
| J8RXI0 | E-value: 170E-39; | Score: 350; | Ident.: 44.1% | unreviewed |
| C2YVQ8 | E-value: 170E-39; | Score: 350; | Ident.: 44.6% | unreviewed |
| A0A1M7GZW8 | E-value: 170E-39; | Score: 350; | Ident.: 44.1% | unreviewed |
| R8LKJ0 | E-value: 170E-39; | Score: 350; | Ident.: 44.1% | unreviewed |
| R8TP71 | E-value: 170E-39; | Score: 350; | Ident.: 44.6% | unreviewed |
| A0A075LHV7 | E-value: 170E-39; | Score: 350; | Ident.: 44.6% | unreviewed |
| R8V567 | E-value: 170E-39; | Score: 350; | Ident.: 44.6% | unreviewed |
| S3JML1 | E-value: 170E-39; | Score: 350; | Ident.: 44.1% | unreviewed |
| K0FXI8 | E-value: 170E-39; | Score: 350; | Ident.: 44.1% | unreviewed |
| A0A1J9YXE5 | E-value: 170E-39; | Score: 350; | Ident.: 44.6% | unreviewed |
| A0A1G4L3M2 | E-value: 170E-39; | Score: 350; | Ident.: 44.6% | unreviewed |
| A0A1J9XI20 | E-value: 170E-39; | Score: 350; | Ident.: 44.1% | unreviewed |
| Q635V4 | E-value: 240E-39; | Score: 349; | Ident.: 44.6% | unreviewed |
| A0A1K0B537 | E-value: 240E-39; | Score: 349; | Ident.: 45.2% | unreviewed |
| A0A161QHG5 | E-value: 240E-39; | Score: 349; | Ident.: 44.6% | unreviewed |
| A0A1B3XIE0 | E-value: 290E-39; | Score: 349; | Ident.: 44.2% | unreviewed |
| C3E7R5 | E-value: 310E-39; | Score: 348; | Ident.: 44.1% | unreviewed |
| C3H5G8 | E-value: 310E-39; | Score: 348; | Ident.: 44.1% | unreviewed |
| C2WRZ8 | E-value: 310E-39; | Score: 348; | Ident.: 44.1% | unreviewed |
| J8IZ88 | E-value: 330E-39; | Score: 348; | Ident.: 44.1% | unreviewed |
| J8MAX8 | E-value: 330E-39; | Score: 348; | Ident.: 44.1% | unreviewed |
| A0A023P998 | E-value: 330E-39; | Score: 348; | Ident.: 44.1% | unreviewed |
| J7VYD3 | E-value: 330E-39; | Score: 348; | Ident.: 44.1% | unreviewed |
| M1QR43 | E-value: 330E-39; | Score: 348; | Ident.: 44.1% | unreviewed |
| A0A0F6JGF2 | E-value: 330E-39; | Score: 348; | Ident.: 44.1% | unreviewed |
| A0A1M6J1Z4 | E-value: 330E-39; | Score: 348; | Ident.: 44.1% | unreviewed |
| C2N5J6 | E-value: 330E-39; | Score: 348; | Ident.: 44.1% | unreviewed |
| R8LE35 | E-value: 330E-39; | Score: 348; | Ident.: 44.1% | unreviewed |
| A0A0W7YEU4 | E-value: 330E-39; | Score: 348; | Ident.: 44.1% | unreviewed |
| C2NM28 | E-value: 330E-39; | Score: 348; | Ident.: 44.6% | unreviewed |
| A0A0D0R0Z7 | E-value: 330E-39; | Score: 348; | Ident.: 44.1% | unreviewed |
| A0A1Q9KH51 | E-value: 330E-39; | Score: 348; | Ident.: 44.1% | unreviewed |
| A0A193CWN0 | E-value: 330E-39; | Score: 348; | Ident.: 44.1% | unreviewed |
| R8GC69 | E-value: 330E-39; | Score: 348; | Ident.: 44.1% | unreviewed |
| R8GUD8 | E-value: 330E-39; | Score: 348; | Ident.: 44.1% | unreviewed |
| R8DZ24 | E-value: 330E-39; | Score: 348; | Ident.: 44.1% | unreviewed |
| J9CDR7 | E-value: 330E-39; | Score: 348; | Ident.: 44.1% | unreviewed |
| V5MEM6 | E-value: 330E-39; | Score: 348; | Ident.: 44.1% | unreviewed |
| J8MQZ4 | E-value: 330E-39; | Score: 348; | Ident.: 44.1% | unreviewed |
| C2XFY3 | E-value: 330E-39; | Score: 348; | Ident.: 44.1% | unreviewed |
| R8FGB5 | E-value: 330E-39; | Score: 348; | Ident.: 44.1% | unreviewed |
| C2T578 | E-value: 330E-39; | Score: 348; | Ident.: 44.1% | unreviewed |
| A0A0D1QWH2 | E-value: 330E-39; | Score: 348; | Ident.: 44.1% | unreviewed |
| A0A0D0QPE4 | E-value: 330E-39; | Score: 348; | Ident.: 44.1% | unreviewed |
| A0A1J9V6H7 | E-value: 330E-39; | Score: 348; | Ident.: 44.1% | unreviewed |
| W8Y7N6 | E-value: 330E-39; | Score: 348; | Ident.: 44.1% | unreviewed |
| A0A1J7S1X1 | E-value: 330E-39; | Score: 348; | Ident.: 44.1% | unreviewed |
| A0A0K0SCM3 | E-value: 330E-39; | Score: 348; | Ident.: 44.1% | unreviewed |
| J8H1U8 | E-value: 330E-39; | Score: 348; | Ident.: 44.1% | unreviewed |
| A0A0G4CYA5 | E-value: 330E-39; | Score: 348; | Ident.: 44.1% | unreviewed |
| J8HQT7 | E-value: 330E-39; | Score: 348; | Ident.: 44.1% | unreviewed |
| R8PIX1 | E-value: 330E-39; | Score: 348; | Ident.: 44.1% | unreviewed |
| R8G100 | E-value: 330E-39; | Score: 348; | Ident.: 44.1% | unreviewed |
| A0A1G1URU5 | E-value: 330E-39; | Score: 348; | Ident.: 44.1% | unreviewed |
| J8ILR3 | E-value: 330E-39; | Score: 348; | Ident.: 44.1% | unreviewed |
| R8E7Z7 | E-value: 330E-39; | Score: 348; | Ident.: 44.1% | unreviewed |
| R8K4B5 | E-value: 330E-39; | Score: 348; | Ident.: 44.1% | unreviewed |
| G9Q2J7 | E-value: 330E-39; | Score: 348; | Ident.: 44.1% | unreviewed |
| C2RCK0 | E-value: 330E-39; | Score: 348; | Ident.: 44.1% | unreviewed |
| J8MNM8 | E-value: 330E-39; | Score: 348; | Ident.: 44.1% | unreviewed |
| C2UIJ6 | E-value: 330E-39; | Score: 348; | Ident.: 44.1% | unreviewed |
| R8TIM0 | E-value: 330E-39; | Score: 348; | Ident.: 44.1% | unreviewed |
| C3FPM5 | E-value: 330E-39; | Score: 348; | Ident.: 44.1% | unreviewed |
| A0A135WV49 | E-value: 330E-39; | Score: 348; | Ident.: 44.1% | unreviewed |
| A0A158RQ70 | E-value: 330E-39; | Score: 348; | Ident.: 44.6% | unreviewed |
| A0A0T8I1T8 | E-value: 330E-39; | Score: 348; | Ident.: 44.1% | unreviewed |
| A0A0F6FQS8 | E-value: 330E-39; | Score: 348; | Ident.: 44.1% | unreviewed |
| A0A0G8DNR2 | E-value: 330E-39; | Score: 348; | Ident.: 44.1% | unreviewed |
| A0A0G3E3F5 | E-value: 330E-39; | Score: 348; | Ident.: 44.1% | unreviewed |
| A0A1C9BXT9 | E-value: 330E-39; | Score: 348; | Ident.: 44.1% | unreviewed |
| Q819K2 | E-value: 330E-39; | Score: 348; | Ident.: 44.1% | reviewed |
| C2P2W6 | E-value: 470E-39; | Score: 347; | Ident.: 43.5% | unreviewed |
| A0A1K0B9C5 | E-value: 470E-39; | Score: 347; | Ident.: 44.1% | unreviewed |
| A0A150DJD9 | E-value: 470E-39; | Score: 347; | Ident.: 44.1% | unreviewed |
| C3I595 | E-value: 470E-39; | Score: 347; | Ident.: 44.1% | unreviewed |
| A0A0R2DT11 | E-value: 470E-39; | Score: 347; | Ident.: 43.2% | unreviewed |
| A0A1H6BVW3 | E-value: 470E-39; | Score: 347; | Ident.: 44.1% | unreviewed |
| A0A1B1SUL9 | E-value: 660E-39; | Score: 346; | Ident.: 44.1% | unreviewed |
| A0A1R1F6X6 | E-value: 810E-39; | Score: 346; | Ident.: 43.2% | unreviewed |
| A0A1S1YLJ7 | E-value: 810E-39; | Score: 346; | Ident.: 43.3% | unreviewed |
| A0A160MGF9 | E-value: 810E-39; | Score: 346; | Ident.: 43.3% | unreviewed |
| R8LD81 | E-value: 930E-39; | Score: 345; | Ident.: 44.6% | unreviewed |
| A0A1J9VVU5 | E-value: 930E-39; | Score: 345; | Ident.: 44.0% | unreviewed |
| J8F5K6 | E-value: 930E-39; | Score: 345; | Ident.: 44.6% | unreviewed |
| B7H6W1 | E-value: 930E-39; | Score: 345; | Ident.: 43.5% | unreviewed |
| A0A0J6NY97 | E-value: 930E-39; | Score: 345; | Ident.: 44.0% | unreviewed |
| A0A0J6Z462 | E-value: 930E-39; | Score: 345; | Ident.: 43.5% | unreviewed |
| A0A1B1LAT3 | E-value: 930E-39; | Score: 345; | Ident.: 44.1% | unreviewed |
| A0A161T8L8 | E-value: 930E-39; | Score: 345; | Ident.: 44.1% | unreviewed |
| R8Q4K1 | E-value: 930E-39; | Score: 345; | Ident.: 44.6% | unreviewed |
| A0A1C4F8A4 | E-value: 930E-39; | Score: 345; | Ident.: 44.6% | unreviewed |
| A0A0Q3TM60 | E-value: 1.2E-36; | Score: 345; | Ident.: 43.6% | unreviewed |
| C2QGA6 | E-value: 1.2E-36; | Score: 344; | Ident.: 44.6% | unreviewed |
| C2VG38 | E-value: 1.2E-36; | Score: 344; | Ident.: 43.5% | unreviewed |
| A0A0N0M862 | E-value: 1.3E-36; | Score: 344; | Ident.: 46.6% | unreviewed |
| C2UZQ0 | E-value: 1.3E-36; | Score: 344; | Ident.: 43.5% | unreviewed |
| A0A1Q5QGP8 | E-value: 1.3E-36; | Score: 344; | Ident.: 43.5% | unreviewed |
| A0A0L1NF76 | E-value: 1.3E-36; | Score: 344; | Ident.: 43.5% | unreviewed |
| J8JRZ0 | E-value: 1.3E-36; | Score: 344; | Ident.: 44.0% | unreviewed |
| A0A1D3PM98 | E-value: 1.3E-36; | Score: 344; | Ident.: 43.5% | unreviewed |
| A0A089LSY6 | E-value: 1.4E-36; | Score: 344; | Ident.: 43.6% | unreviewed |
| A0A150L806 | E-value: 1.6E-36; | Score: 344; | Ident.: 46.8% | unreviewed |
| A0A0K9GNZ1 | E-value: 1.6E-36; | Score: 344; | Ident.: 42.0% | unreviewed |
| E5WFK1 | E-value: 1.6E-36; | Score: 344; | Ident.: 43.3% | unreviewed |
| A0A0Q3RBL2 | E-value: 1.7E-36; | Score: 344; | Ident.: 42.9% | unreviewed |
| A0A0T6BPJ5 | E-value: 1.7E-36; | Score: 344; | Ident.: 44.0% | unreviewed |
| A0A0M0WY71 | E-value: 1.7E-36; | Score: 344; | Ident.: 42.9% | unreviewed |
| G1VN14 | E-value: 1.8E-36; | Score: 343; | Ident.: 41.0% | unreviewed |
| N9WD85 | E-value: 1.8E-36; | Score: 343; | Ident.: 41.0% | unreviewed |
| B1C397 | E-value: 2.1E-36; | Score: 343; | Ident.: 43.6% | unreviewed |
| W7KW66 | E-value: 2.3E-36; | Score: 343; | Ident.: 42.7% | unreviewed |
| A0A0J5W8D9 | E-value: 2.3E-36; | Score: 343; | Ident.: 42.2% | unreviewed |
| C2SPC4 | E-value: 2.4E-36; | Score: 342; | Ident.: 44.0% | unreviewed |
| J8AG13 | E-value: 2.6E-36; | Score: 342; | Ident.: 44.0% | unreviewed |
| J7TE35 | E-value: 2.6E-36; | Score: 342; | Ident.: 44.0% | unreviewed |
| J8P1E4 | E-value: 2.6E-36; | Score: 342; | Ident.: 44.0% | unreviewed |
| R8D4S4 | E-value: 2.6E-36; | Score: 342; | Ident.: 44.0% | unreviewed |
| J8CWL8 | E-value: 2.6E-36; | Score: 342; | Ident.: 44.0% | unreviewed |
| C2Q023 | E-value: 2.6E-36; | Score: 342; | Ident.: 44.0% | unreviewed |
| J8F5G3 | E-value: 2.6E-36; | Score: 342; | Ident.: 44.0% | unreviewed |
| J8J0P7 | E-value: 2.6E-36; | Score: 342; | Ident.: 44.0% | unreviewed |
| R8I456 | E-value: 2.6E-36; | Score: 342; | Ident.: 44.0% | unreviewed |
| J9C9E8 | E-value: 2.6E-36; | Score: 342; | Ident.: 44.0% | unreviewed |
| R8CWI7 | E-value: 2.6E-36; | Score: 342; | Ident.: 44.0% | unreviewed |
| W4DSF5 | E-value: 2.6E-36; | Score: 342; | Ident.: 44.0% | unreviewed |
| A0A1H6QLE2 | E-value: 2.6E-36; | Score: 342; | Ident.: 42.9% | unreviewed |
| J9B5H6 | E-value: 2.6E-36; | Score: 342; | Ident.: 44.0% | unreviewed |
| A0A1K0AEU2 | E-value: 2.6E-36; | Score: 342; | Ident.: 44.0% | unreviewed |
| C2XYE3 | E-value: 2.6E-36; | Score: 342; | Ident.: 44.0% | unreviewed |
| R8MM75 | E-value: 2.6E-36; | Score: 342; | Ident.: 44.0% | unreviewed |
| R8EMT0 | E-value: 2.6E-36; | Score: 342; | Ident.: 44.0% | unreviewed |
| B5USC2 | E-value: 2.6E-36; | Score: 342; | Ident.: 43.4% | unreviewed |

**BLAST searches (tab file format) for PDF in *Mycoplasma bovis* showing no *M. hyopneumoniae* matches.**

| **Entry** | **Info** |  |  | **Status** |
| --- | --- | --- | --- | --- |
| E4Q0J2 | E-value: 5.0E-129; | Score: 956; | Ident.: 100.0% | unreviewed |
| A0A059Y7Z0 | E-value: 5.0E-129; | Score: 956; | Ident.: 100.0% | unreviewed |
| I5D5M9 | E-value: 570E-111; | Score: 824; | Ident.: 84.4% | unreviewed |
| A5IXW9 | E-value: 6.6E-108; | Score: 817; | Ident.: 83.9% | unreviewed |
| D3VPL5 | E-value: 110E-108; | Score: 809; | Ident.: 83.3% | unreviewed |
| D5E4Q1 | E-value: 12E-75; | Score: 597; | Ident.: 66.1% | unreviewed |
| D4XWB8 | E-value: 560E-75; | Score: 586; | Ident.: 66.1% | unreviewed |
| C4XE56 | E-value: 4.7E-72; | Score: 581; | Ident.: 65.7% | unreviewed |
| A0A059XMQ5 | E-value: 110E-72; | Score: 572; | Ident.: 61.9% | unreviewed |
| A0A060PYH4 | E-value: 110E-72; | Score: 572; | Ident.: 61.9% | unreviewed |
| F9QDM4 | E-value: 3.2E-69; | Score: 561; | Ident.: 63.1% | unreviewed |
| A0A1L4FSH9 | E-value: 37E-69; | Score: 554; | Ident.: 64.4% | unreviewed |
| N9TUH4 | E-value: 110E-69; | Score: 552; | Ident.: 58.6% | unreviewed |
| A0A1L7MYC6 | E-value: 110E-69; | Score: 552; | Ident.: 58.6% | unreviewed |
| A0A0D5ZJP8 | E-value: 490E-69; | Score: 547; | Ident.: 61.2% | unreviewed |
| Q98PN3 | E-value: 2.8E-66; | Score: 543; | Ident.: 58.3% | reviewed |
| I1A7I5 | E-value: 5.6E-66; | Score: 540; | Ident.: 55.9% | unreviewed |
| A0A0F6ZLM2 | E-value: 16E-66; | Score: 537; | Ident.: 55.9% | unreviewed |
| Q6KH28 | E-value: 41E-66; | Score: 534; | Ident.: 57.9% | unreviewed |
| N9TRB3 | E-value: 120E-66; | Score: 531; | Ident.: 56.9% | unreviewed |
| N9UAD5 | E-value: 170E-66; | Score: 530; | Ident.: 56.2% | unreviewed |
| L0RVV9 | E-value: 360E-66; | Score: 528; | Ident.: 55.6% | unreviewed |
| A0A097NSQ3 | E-value: 670E-66; | Score: 526; | Ident.: 55.5% | unreviewed |
| D1J7P7 | E-value: 670E-66; | Score: 526; | Ident.: 55.5% | unreviewed |
| A0A168RJL0 | E-value: 1.3E-63; | Score: 524; | Ident.: 58.9% | unreviewed |
| A0A063YAC7 | E-value: 8.4E-63; | Score: 519; | Ident.: 58.6% | unreviewed |
| A0A077L790 | E-value: 95E-63; | Score: 512; | Ident.: 53.9% | unreviewed |
| A0A0C6G2F0 | E-value: 2.0E-60; | Score: 503; | Ident.: 52.8% | unreviewed |
| B3PN62 | E-value: 63E-60; | Score: 493; | Ident.: 51.6% | unreviewed |
| A0A0E3H7N1 | E-value: 140E-60; | Score: 491; | Ident.: 56.9% | unreviewed |
| Q4A730 | E-value: 800E-60; | Score: 486; | Ident.: 56.3% | unreviewed |
| F9UJ31 | E-value: 1.4E-57; | Score: 485; | Ident.: 58.4% | unreviewed |
| A0A0F5H121 | E-value: 1.9E-54; | Score: 464; | Ident.: 55.1% | unreviewed |
| A0A0R1YA58 | E-value: 34E-33; | Score: 314; | Ident.: 43.2% | unreviewed |
| A0A0X8H2A4 | E-value: 53E-33; | Score: 313; | Ident.: 38.4% | unreviewed |
| G5KA60 | E-value: 150E-33; | Score: 311; | Ident.: 39.7% | unreviewed |
| F3L731 | E-value: 430E-33; | Score: 308; | Ident.: 39.2% | unreviewed |
| A0A0N0CPM7 | E-value: 560E-33; | Score: 306; | Ident.: 39.8% | unreviewed |
| A0A0N1CLT7 | E-value: 780E-33; | Score: 305; | Ident.: 37.6% | unreviewed |
| A0A0E9DCV7 | E-value: 900E-33; | Score: 299; | Ident.: 50.4% | unreviewed |
| A0A0R1XZU2 | E-value: 1.1E-30; | Score: 304; | Ident.: 42.3% | unreviewed |
| A0A0X8PNY6 | E-value: 1.1E-30; | Score: 304; | Ident.: 37.6% | unreviewed |
| R2PIJ6 | E-value: 1.1E-30; | Score: 304; | Ident.: 37.6% | unreviewed |
| T2NSH6 | E-value: 1.1E-30; | Score: 304; | Ident.: 37.6% | unreviewed |
| C9CKW5 | E-value: 1.1E-30; | Score: 304; | Ident.: 37.6% | unreviewed |
| S4BBC2 | E-value: 1.1E-30; | Score: 304; | Ident.: 37.6% | unreviewed |
| J0XQV6 | E-value: 1.1E-30; | Score: 304; | Ident.: 37.6% | unreviewed |
| S4D4L5 | E-value: 1.1E-30; | Score: 304; | Ident.: 37.6% | unreviewed |
| C9A9T0 | E-value: 1.1E-30; | Score: 304; | Ident.: 37.6% | unreviewed |
| A0A1L8SFF2 | E-value: 1.1E-30; | Score: 304; | Ident.: 37.6% | unreviewed |
| Q48661 | E-value: 1.9E-30; | Score: 303; | Ident.: 37.3% | reviewed |
| A0A199YT22 | E-value: 2.4E-30; | Score: 303; | Ident.: 37.3% | unreviewed |
| T0WN17 | E-value: 2.7E-30; | Score: 303; | Ident.: 37.3% | unreviewed |
| T0UCA8 | E-value: 2.7E-30; | Score: 303; | Ident.: 37.3% | unreviewed |
| H5SX86 | E-value: 2.7E-30; | Score: 303; | Ident.: 37.3% | unreviewed |
| T0RVZ0 | E-value: 2.7E-30; | Score: 303; | Ident.: 37.3% | unreviewed |
| A0A161V0S5 | E-value: 2.7E-30; | Score: 303; | Ident.: 37.3% | unreviewed |
| Q031G9 | E-value: 2.7E-30; | Score: 303; | Ident.: 37.3% | unreviewed |
| A0A1L9C071 | E-value: 2.7E-30; | Score: 303; | Ident.: 37.3% | unreviewed |
| G8P7J8 | E-value: 2.7E-30; | Score: 303; | Ident.: 37.3% | unreviewed |
| U5PJ71 | E-value: 2.7E-30; | Score: 303; | Ident.: 37.3% | unreviewed |
| K7W7B9 | E-value: 2.7E-30; | Score: 303; | Ident.: 37.3% | unreviewed |
| A0A084AC11 | E-value: 2.7E-30; | Score: 303; | Ident.: 37.3% | unreviewed |
| S6FLG1 | E-value: 2.7E-30; | Score: 303; | Ident.: 37.3% | unreviewed |
| A2RIP1 | E-value: 2.7E-30; | Score: 303; | Ident.: 37.3% | unreviewed |
| D2BP29 | E-value: 2.7E-30; | Score: 303; | Ident.: 37.3% | unreviewed |
| G6FCB2 | E-value: 2.7E-30; | Score: 303; | Ident.: 37.3% | unreviewed |
| A0A1E7G1H8 | E-value: 2.7E-30; | Score: 303; | Ident.: 37.3% | unreviewed |
| U6EQH5 | E-value: 2.7E-30; | Score: 303; | Ident.: 37.3% | unreviewed |
| T2F454 | E-value: 2.7E-30; | Score: 303; | Ident.: 37.3% | unreviewed |
| A0A0A7SZZ8 | E-value: 2.7E-30; | Score: 303; | Ident.: 37.3% | unreviewed |
| A0A0M2ZUH5 | E-value: 2.7E-30; | Score: 303; | Ident.: 37.3% | unreviewed |
| A0A166ZEK9 | E-value: 2.7E-30; | Score: 303; | Ident.: 37.3% | unreviewed |
| A0A0R2EFT3 | E-value: 3.2E-30; | Score: 301; | Ident.: 41.6% | unreviewed |
| A0A142KQI2 | E-value: 3.2E-30; | Score: 301; | Ident.: 41.6% | unreviewed |
| G5JZR9 | E-value: 3.3E-30; | Score: 302; | Ident.: 38.2% | unreviewed |
| A0A089XPE8 | E-value: 3.8E-30; | Score: 302; | Ident.: 37.3% | unreviewed |
| A0A0R2BYV1 | E-value: 3.9E-30; | Score: 300; | Ident.: 42.0% | unreviewed |
| A0A0R1S9M8 | E-value: 4.0E-30; | Score: 300; | Ident.: 39.9% | unreviewed |
| A0A0R2LPG6 | E-value: 4.3E-30; | Score: 300; | Ident.: 40.7% | unreviewed |
| A0A0E2EPT3 | E-value: 4.6E-30; | Score: 301; | Ident.: 38.7% | unreviewed |
| A8R7Q1 | E-value: 5.7E-30; | Score: 299; | Ident.: 37.0% | unreviewed |
| A0A0G6PMY4 | E-value: 6.4E-30; | Score: 300; | Ident.: 38.6% | unreviewed |
| A0A0D1AEW0 | E-value: 6.4E-30; | Score: 300; | Ident.: 38.6% | unreviewed |
| A0A0D1BBJ2 | E-value: 6.4E-30; | Score: 300; | Ident.: 38.6% | unreviewed |
| A0A0E1DVI0 | E-value: 6.4E-30; | Score: 300; | Ident.: 38.6% | unreviewed |
| A0A0D1AVD8 | E-value: 6.4E-30; | Score: 300; | Ident.: 38.6% | unreviewed |
| A0A072ELI0 | E-value: 6.4E-30; | Score: 300; | Ident.: 38.6% | unreviewed |
| A0A1N6TL15 | E-value: 6.4E-30; | Score: 300; | Ident.: 38.6% | unreviewed |
| B4U576 | E-value: 6.4E-30; | Score: 300; | Ident.: 38.6% | reviewed |
| C0M9E1 | E-value: 6.4E-30; | Score: 300; | Ident.: 38.6% | reviewed |
| A0A0R2ASY0 | E-value: 7.6E-30; | Score: 298; | Ident.: 40.7% | unreviewed |
| A0A1E8GHU1 | E-value: 7.6E-30; | Score: 299; | Ident.: 37.8% | unreviewed |
| A0A0D4CLT1 | E-value: 8.2E-30; | Score: 298; | Ident.: 40.6% | unreviewed |
| A0A0R1NZ08 | E-value: 8.2E-30; | Score: 298; | Ident.: 40.6% | unreviewed |
| G2KWJ3 | E-value: 8.2E-30; | Score: 298; | Ident.: 42.0% | unreviewed |
| A0A099YC93 | E-value: 8.2E-30; | Score: 298; | Ident.: 40.6% | unreviewed |
| F0EGA3 | E-value: 8.4E-30; | Score: 298; | Ident.: 37.0% | unreviewed |
| A0A0P7K321 | E-value: 8.4E-30; | Score: 298; | Ident.: 39.2% | unreviewed |
| A0A0C3AGR3 | E-value: 8.4E-30; | Score: 298; | Ident.: 39.2% | unreviewed |
| A0A0R1FRK7 | E-value: 11E-30; | Score: 297; | Ident.: 39.2% | unreviewed |
| A0A0M9D8E3 | E-value: 12E-30; | Score: 297; | Ident.: 39.2% | unreviewed |
| A0A087EP89 | E-value: 12E-30; | Score: 297; | Ident.: 39.2% | unreviewed |
| A0A1D7ZYH4 | E-value: 12E-30; | Score: 297; | Ident.: 41.0% | unreviewed |
| A0A139N967 | E-value: 13E-30; | Score: 298; | Ident.: 37.7% | unreviewed |
| A0A075STM0 | E-value: 13E-30; | Score: 298; | Ident.: 36.6% | unreviewed |
| C0MFA6 | E-value: 13E-30; | Score: 298; | Ident.: 38.6% | reviewed |
| T0WDU3 | E-value: 15E-30; | Score: 298; | Ident.: 36.8% | unreviewed |
| A0A0V8DFX5 | E-value: 15E-30; | Score: 298; | Ident.: 36.8% | unreviewed |
| A0A1B9AT28 | E-value: 15E-30; | Score: 296; | Ident.: 39.2% | unreviewed |
| A0A0N8IGS9 | E-value: 16E-30; | Score: 296; | Ident.: 38.6% | unreviewed |
| A0A0Z8PT80 | E-value: 18E-30; | Score: 297; | Ident.: 37.2% | unreviewed |
| A0A0Z8UEU2 | E-value: 18E-30; | Score: 297; | Ident.: 37.2% | unreviewed |
| A0A1L7LGS2 | E-value: 18E-30; | Score: 297; | Ident.: 38.2% | unreviewed |
| C2ERY1 | E-value: 18E-30; | Score: 296; | Ident.: 39.2% | unreviewed |
| A0A0R1X5B8 | E-value: 18E-30; | Score: 296; | Ident.: 40.4% | unreviewed |
| A0A0R1VXW8 | E-value: 21E-30; | Score: 296; | Ident.: 40.3% | unreviewed |
| R9WLB6 | E-value: 23E-30; | Score: 295; | Ident.: 41.2% | unreviewed |
| F8KC85 | E-value: 23E-30; | Score: 295; | Ident.: 41.2% | unreviewed |
| A0A0S4NPF2 | E-value: 23E-30; | Score: 295; | Ident.: 41.2% | unreviewed |
| A0A0Z8H5P3 | E-value: 25E-30; | Score: 296; | Ident.: 37.2% | unreviewed |
| A0A0Z8S7J1 | E-value: 25E-30; | Score: 296; | Ident.: 36.6% | unreviewed |
| A0A168Z183 | E-value: 25E-30; | Score: 296; | Ident.: 36.6% | unreviewed |
| A0A0R1W3V1 | E-value: 27E-30; | Score: 295; | Ident.: 41.2% | unreviewed |
| C8P7R7 | E-value: 29E-30; | Score: 295; | Ident.: 40.4% | unreviewed |
| A0A0R2GFZ2 | E-value: 29E-30; | Score: 294; | Ident.: 42.0% | unreviewed |
| A0A1F0NKB3 | E-value: 34E-30; | Score: 295; | Ident.: 38.4% | unreviewed |
| T0TTK9 | E-value: 34E-30; | Score: 294; | Ident.: 40.5% | unreviewed |
| A0A0E2EGI0 | E-value: 35E-30; | Score: 295; | Ident.: 37.6% | unreviewed |
| Q8DWC2 | E-value: 35E-30; | Score: 295; | Ident.: 37.6% | reviewed |
| A0A0R1IIF6 | E-value: 41E-30; | Score: 293; | Ident.: 41.4% | unreviewed |
| A0A0R1W893 | E-value: 41E-30; | Score: 293; | Ident.: 40.4% | unreviewed |
| A0A142KP76 | E-value: 44E-30; | Score: 293; | Ident.: 40.4% | unreviewed |
| B3XRL7 | E-value: 44E-30; | Score: 293; | Ident.: 41.2% | unreviewed |
| E3C686 | E-value: 44E-30; | Score: 293; | Ident.: 40.4% | unreviewed |
| A0A0C1PMY6 | E-value: 44E-30; | Score: 293; | Ident.: 41.4% | unreviewed |
| A0A073JRF2 | E-value: 44E-30; | Score: 293; | Ident.: 41.2% | unreviewed |
| A0A081NQ91 | E-value: 44E-30; | Score: 293; | Ident.: 41.2% | unreviewed |
| A0A1L8U495 | E-value: 45E-30; | Score: 293; | Ident.: 36.5% | unreviewed |
| C9A282 | E-value: 45E-30; | Score: 293; | Ident.: 36.5% | unreviewed |
| G5IPW0 | E-value: 45E-30; | Score: 293; | Ident.: 36.5% | unreviewed |
| T0VIF4 | E-value: 45E-30; | Score: 293; | Ident.: 36.5% | unreviewed |
| A0A0N0CQ47 | E-value: 45E-30; | Score: 293; | Ident.: 38.6% | unreviewed |
| R4RJJ4 | E-value: 48E-30; | Score: 293; | Ident.: 40.5% | unreviewed |
| V4XLF0 | E-value: 48E-30; | Score: 293; | Ident.: 40.5% | unreviewed |
| D0DVD6 | E-value: 48E-30; | Score: 293; | Ident.: 40.5% | unreviewed |
| A0A0N7CHT5 | E-value: 48E-30; | Score: 293; | Ident.: 40.5% | unreviewed |
| A0A0F4HAU8 | E-value: 48E-30; | Score: 293; | Ident.: 40.5% | unreviewed |
| A0A1Q2T434 | E-value: 48E-30; | Score: 293; | Ident.: 40.5% | unreviewed |
| A0A1F1T5S9 | E-value: 48E-30; | Score: 293; | Ident.: 40.5% | unreviewed |
| B2GBA3 | E-value: 48E-30; | Score: 293; | Ident.: 40.5% | reviewed |
| A0A116LDL7 | E-value: 49E-30; | Score: 294; | Ident.: 36.6% | unreviewed |
| G7SIL0 | E-value: 49E-30; | Score: 294; | Ident.: 36.6% | unreviewed |
| A0A0Z8DK73 | E-value: 49E-30; | Score: 294; | Ident.: 36.6% | unreviewed |
| A0A1E8GMK5 | E-value: 58E-30; | Score: 293; | Ident.: 37.8% | unreviewed |
| R7IIC5 | E-value: 59E-30; | Score: 292; | Ident.: 39.9% | unreviewed |
| F5W1Z5 | E-value: 66E-30; | Score: 293; | Ident.: 38.4% | unreviewed |
| C0WXR8 | E-value: 67E-30; | Score: 292; | Ident.: 39.9% | unreviewed |
| G5JV40 | E-value: 68E-30; | Score: 293; | Ident.: 38.3% | unreviewed |
| A0A0Z8AF88 | E-value: 68E-30; | Score: 293; | Ident.: 36.6% | unreviewed |
| A0A0Z8E5W4 | E-value: 68E-30; | Score: 293; | Ident.: 36.6% | unreviewed |
| A0A0U3DWB0 | E-value: 68E-30; | Score: 293; | Ident.: 39.2% | unreviewed |
| U5UKU2 | E-value: 68E-30; | Score: 293; | Ident.: 36.6% | unreviewed |
| A0A0N0VBU0 | E-value: 68E-30; | Score: 293; | Ident.: 36.6% | unreviewed |
| A0A116KT90 | E-value: 68E-30; | Score: 293; | Ident.: 36.6% | unreviewed |
| B9DTD6 | E-value: 68E-30; | Score: 293; | Ident.: 36.6% | reviewed |
| A0A0R2EAS1 | E-value: 81E-30; | Score: 291; | Ident.: 41.4% | unreviewed |
| A0A0R1VBG1 | E-value: 85E-30; | Score: 291; | Ident.: 42.0% | unreviewed |
| A0A0R1JDV5 | E-value: 87E-30; | Score: 291; | Ident.: 40.7% | unreviewed |
| A0A0F4LUD0 | E-value: 89E-30; | Score: 291; | Ident.: 41.1% | unreviewed |
| A0A0B8TIQ6 | E-value: 94E-30; | Score: 291; | Ident.: 40.5% | unreviewed |
| F8DHH6 | E-value: 95E-30; | Score: 292; | Ident.: 37.6% | unreviewed |
| A0A0F3H2W5 | E-value: 95E-30; | Score: 292; | Ident.: 37.6% | unreviewed |
| V8B9V1 | E-value: 95E-30; | Score: 292; | Ident.: 37.6% | unreviewed |
| S5RIX8 | E-value: 95E-30; | Score: 292; | Ident.: 38.7% | unreviewed |
| A0A0W7UZM6 | E-value: 95E-30; | Score: 292; | Ident.: 38.7% | unreviewed |
| A0A1E8UJK5 | E-value: 95E-30; | Score: 292; | Ident.: 37.6% | unreviewed |
| A0A1F0Y1N1 | E-value: 95E-30; | Score: 292; | Ident.: 37.6% | unreviewed |
| U5PA60 | E-value: 95E-30; | Score: 292; | Ident.: 36.6% | unreviewed |
| A0A1F1CJG1 | E-value: 95E-30; | Score: 292; | Ident.: 37.6% | unreviewed |
| K8MMP3 | E-value: 95E-30; | Score: 292; | Ident.: 37.6% | unreviewed |
| E8K8F2 | E-value: 95E-30; | Score: 292; | Ident.: 37.6% | unreviewed |
| A0A1F0AWM0 | E-value: 95E-30; | Score: 292; | Ident.: 37.6% | unreviewed |
| A0A1F1DJ83 | E-value: 95E-30; | Score: 292; | Ident.: 37.6% | unreviewed |
| T0UX47 | E-value: 95E-30; | Score: 292; | Ident.: 37.6% | unreviewed |
| E3CF52 | E-value: 95E-30; | Score: 292; | Ident.: 37.6% | unreviewed |
| A0A1S1EF39 | E-value: 95E-30; | Score: 292; | Ident.: 37.6% | unreviewed |
| A0A172Q8X8 | E-value: 95E-30; | Score: 292; | Ident.: 34.9% | unreviewed |
| A0A0Z8ER43 | E-value: 95E-30; | Score: 292; | Ident.: 36.6% | unreviewed |
| A0A123SGY4 | E-value: 95E-30; | Score: 292; | Ident.: 36.6% | unreviewed |
| A0A0M9FKE6 | E-value: 95E-30; | Score: 292; | Ident.: 36.6% | unreviewed |
| I1ZK56 | E-value: 95E-30; | Score: 292; | Ident.: 37.6% | unreviewed |
| I2NP60 | E-value: 95E-30; | Score: 292; | Ident.: 37.6% | unreviewed |
| F9M4L0 | E-value: 95E-30; | Score: 292; | Ident.: 37.6% | unreviewed |
| W1V6L8 | E-value: 95E-30; | Score: 292; | Ident.: 37.6% | unreviewed |
| A0A1F0KH62 | E-value: 95E-30; | Score: 292; | Ident.: 37.6% | unreviewed |
| A0A0R2BRD5 | E-value: 110E-30; | Score: 291; | Ident.: 38.6% | unreviewed |
| A0A0C2TWF0 | E-value: 120E-30; | Score: 290; | Ident.: 39.2% | unreviewed |
| A0A0A6NV00 | E-value: 120E-30; | Score: 290; | Ident.: 40.4% | unreviewed |
| A0A0F3RQU6 | E-value: 120E-30; | Score: 290; | Ident.: 40.9% | unreviewed |
| A0A1C1ZCW0 | E-value: 120E-30; | Score: 290; | Ident.: 41.2% | unreviewed |
| H4GL57 | E-value: 130E-30; | Score: 290; | Ident.: 42.0% | unreviewed |
| A0A0R1RAI8 | E-value: 130E-30; | Score: 290; | Ident.: 40.9% | unreviewed |
| A0A1E9B9W8 | E-value: 130E-30; | Score: 291; | Ident.: 38.4% | unreviewed |
| A0A1E8ZUU5 | E-value: 130E-30; | Score: 291; | Ident.: 38.4% | unreviewed |
| I2J078 | E-value: 130E-30; | Score: 291; | Ident.: 37.9% | unreviewed |
| A0A0P6S4R6 | E-value: 130E-30; | Score: 291; | Ident.: 39.3% | unreviewed |
| A0A1E8TUU6 | E-value: 130E-30; | Score: 291; | Ident.: 37.9% | unreviewed |
| A0A0H3MXB0 | E-value: 130E-30; | Score: 291; | Ident.: 36.1% | unreviewed |
| G5L0R0 | E-value: 130E-30; | Score: 291; | Ident.: 36.1% | unreviewed |
| A0A1F0ZRV8 | E-value: 130E-30; | Score: 291; | Ident.: 37.6% | unreviewed |
| A0A0Z8E5N9 | E-value: 130E-30; | Score: 291; | Ident.: 36.1% | unreviewed |
| A0A0Z8HER8 | E-value: 130E-30; | Score: 291; | Ident.: 36.1% | unreviewed |
| G7SP42 | E-value: 130E-30; | Score: 291; | Ident.: 36.6% | unreviewed |
| D5AK48 | E-value: 130E-30; | Score: 291; | Ident.: 36.1% | unreviewed |
| J3AI09 | E-value: 130E-30; | Score: 291; | Ident.: 38.2% | unreviewed |
| A0A0Z8G3J0 | E-value: 130E-30; | Score: 291; | Ident.: 36.1% | unreviewed |
| A4VXS1 | E-value: 130E-30; | Score: 291; | Ident.: 36.1% | reviewed |
| A4W418 | E-value: 130E-30; | Score: 291; | Ident.: 36.1% | reviewed |
| A0A0H4QET4 | E-value: 170E-30; | Score: 289; | Ident.: 40.7% | unreviewed |
| S5NAJ1 | E-value: 170E-30; | Score: 289; | Ident.: 40.6% | unreviewed |
| A0A1C2GBT6 | E-value: 170E-30; | Score: 289; | Ident.: 40.6% | unreviewed |
| X0P9I1 | E-value: 180E-30; | Score: 289; | Ident.: 39.3% | unreviewed |
| A0A1Q8EDG0 | E-value: 190E-30; | Score: 290; | Ident.: 35.1% | unreviewed |
| A0A116S2U8 | E-value: 190E-30; | Score: 290; | Ident.: 36.1% | unreviewed |
| E8JR15 | E-value: 190E-30; | Score: 290; | Ident.: 38.7% | unreviewed |
| A0A0Z8LC95 | E-value: 190E-30; | Score: 290; | Ident.: 35.6% | unreviewed |
| A0A116RGH8 | E-value: 190E-30; | Score: 290; | Ident.: 36.1% | unreviewed |
| A0A075TZV3 | E-value: 210E-30; | Score: 289; | Ident.: 37.4% | unreviewed |
| A0A0R1VCR7 | E-value: 220E-30; | Score: 289; | Ident.: 39.3% | unreviewed |
| A0A0R1RKA9 | E-value: 230E-30; | Score: 288; | Ident.: 42.4% | unreviewed |
| R7P849 | E-value: 230E-30; | Score: 288; | Ident.: 39.9% | unreviewed |
| A0A143WC44 | E-value: 250E-30; | Score: 288; | Ident.: 42.4% | unreviewed |
| A0A135YXL5 | E-value: 260E-30; | Score: 289; | Ident.: 37.7% | unreviewed |
| A0A127TTP2 | E-value: 260E-30; | Score: 289; | Ident.: 37.0% | unreviewed |
| A0A139NNR7 | E-value: 260E-30; | Score: 289; | Ident.: 38.5% | unreviewed |
| E0PBG1 | E-value: 260E-30; | Score: 289; | Ident.: 37.7% | unreviewed |
| F5X4J3 | E-value: 260E-30; | Score: 289; | Ident.: 37.7% | unreviewed |
| A0A0W7V362 | E-value: 260E-30; | Score: 289; | Ident.: 37.7% | unreviewed |
| A0A0M0KIZ8 | E-value: 310E-30; | Score: 287; | Ident.: 40.4% | unreviewed |
| Q9K9I9 | E-value: 310E-30; | Score: 287; | Ident.: 40.4% | reviewed |
| A0A0R1KMV4 | E-value: 310E-30; | Score: 287; | Ident.: 40.3% | unreviewed |
| A0A0R1PR08 | E-value: 310E-30; | Score: 287; | Ident.: 40.3% | unreviewed |
| A0A0R1I3K4 | E-value: 310E-30; | Score: 287; | Ident.: 40.3% | unreviewed |
| A0A1D2JZ87 | E-value: 310E-30; | Score: 287; | Ident.: 36.7% | unreviewed |
| A0A1D2K9P2 | E-value: 310E-30; | Score: 287; | Ident.: 36.7% | unreviewed |
| A0A1D2LH47 | E-value: 310E-30; | Score: 287; | Ident.: 36.7% | unreviewed |
| K8ZKG8 | E-value: 340E-30; | Score: 287; | Ident.: 39.0% | unreviewed |
| F8DNM0 | E-value: 340E-30; | Score: 287; | Ident.: 40.6% | unreviewed |
| A0A0R1SRR2 | E-value: 340E-30; | Score: 287; | Ident.: 40.1% | unreviewed |

# Supplementary Data S5: N-terminal dimethylated peptides detected upstream within 50 amino acids of the predicted start site, either with new iMet retained (5 proteins) or iMet removed (4 proteins).

| **Gene names** | **Protein names** | **Sequence** |
| --- | --- | --- |
| Possible upstream start site (iMet retained) | | |
| MHJ_0191 | 30S ribosomal protein S10 * | *f*.^32^MNTTSIKIKLKSFDHR |
| MHJ_0278 | 30S ribosomal protein S16 | *r*.^9^MGSKFNPFYKIVVADAR |
| MHJ_0181 | 30S ribosomal protein S17 * | *k*.^4^MNNLTLEKKAQTR |
| MHJ_0122 | Pyruvate kinase * | *d*.^4^MKNYISKR |
| MHJ_0036 | PTS system, lichenan-specific IIA component * | *s*.^30^MKKILIGFTNESFR |
| Possible upstream start site (iMet removed) | | |
| MHJ_0073 | 30S ribosomal protein S12 * | m.^27^LYNSLHKKELKLSAPFKR |
| MHJ_0181 | 30S ribosomal protein S17 * | m.^5^NNLTLEKKAQTR |
| MHJ_0149 | Guanylate kinase | m.^6^SKLIILSGPSGVGKGTIESLL LKNKNLLIKLAISATTR |
| MHJ_0036 | PTS system, lichenan-specific IIA component * | m.^31^KKILIGFTNESFR |

* Possible secondary start site as N-terminal peptides were detected downstream (Supplementary Files S1, S2 & S3)

# Supplementary Data S8: Conserved domain analysis using Conserved Domain Database to search for uncharacterised putative proteases in *Mycoplasma hyopneumoniae* (strain J).

Q4A9B9: Trypsin-like serine protease


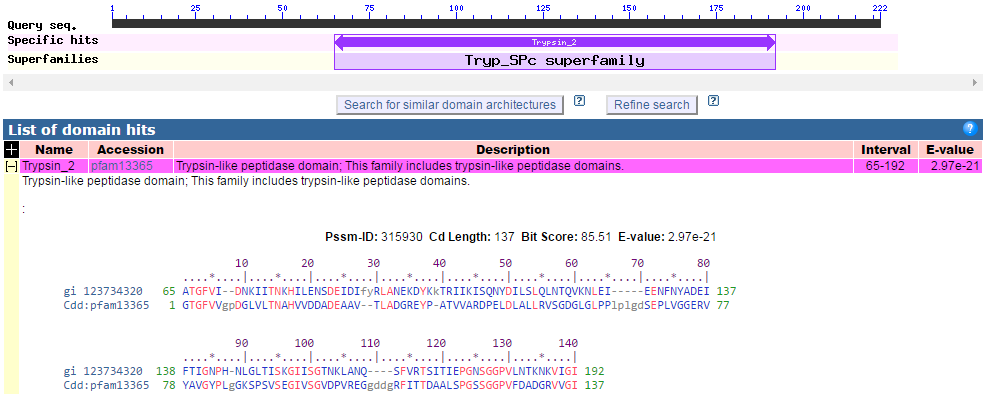


Q4AA00: Subtilisin-like serine protease


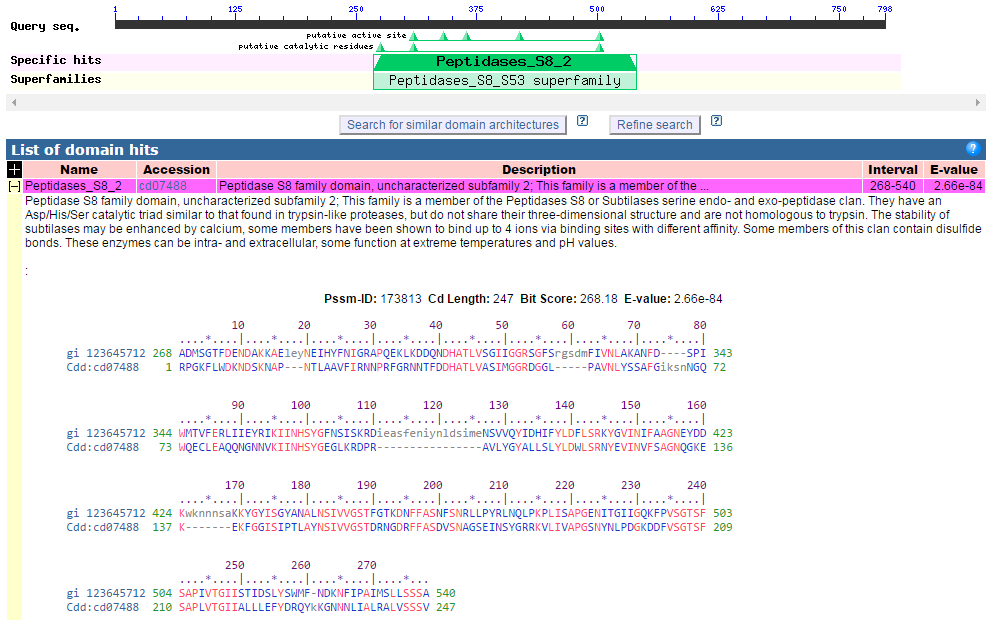


Q4A9T6 Subtilisin-like serine protease


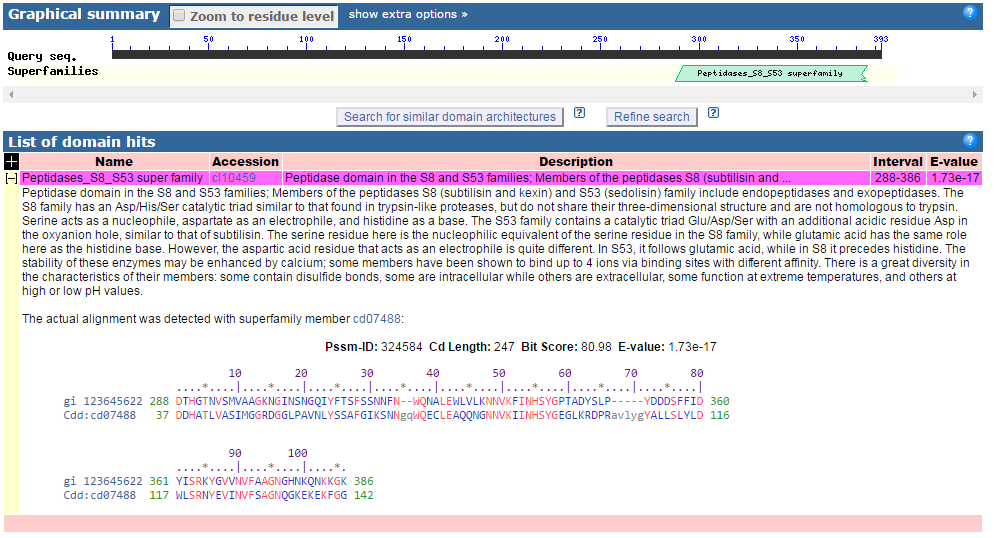


Q4A9I9: Zinc peptidase (M18/M20/M28/M42)


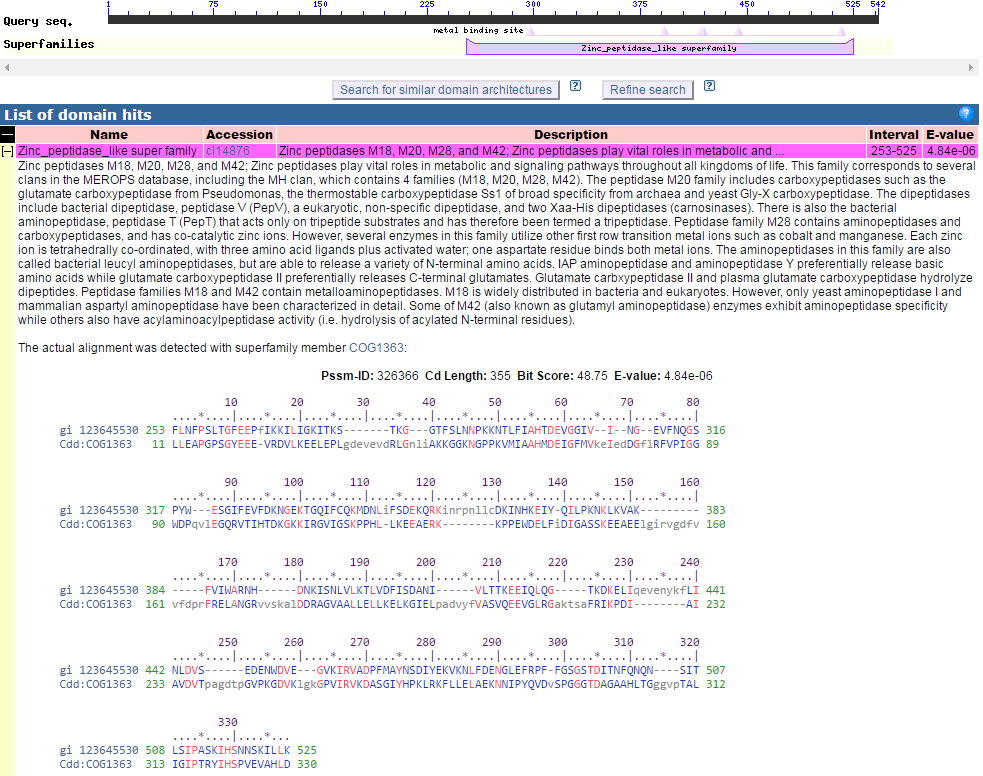


Q4AA40: Peptidase S7

*No conserved domain was found using CDD search, but NCBI sequence viewer (output below) identified a peptidase S7 domain.


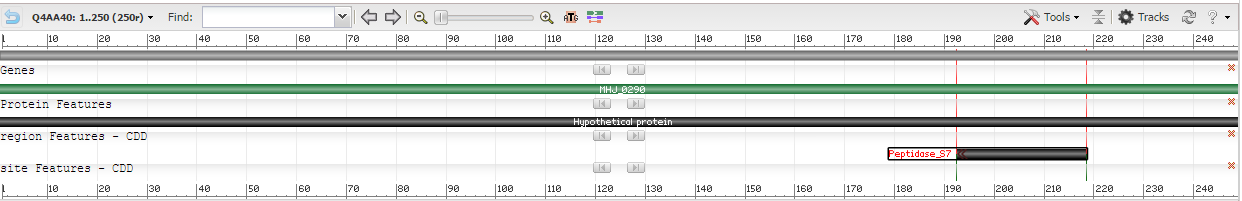


MEROPS description: Peptidase S7 cleaves at sites like -Xaa-Xaa↓Yaa-, where Xaa is normally an amino acid with a basic side chain, and Yaa has a short side chain (Amberg et al., 1994).

# Supplementary Data S9: G3PDH protein:protein interactions predicted by PredictProtein. Only surface exposed protein:protein binding sites shown.

**KEY**P = protein:protein, D = nucleotide:protein, E=both nucleotide: and protein:protein, X=could not assess

**F0 (Full Length)**

MKKIAINGFGRIGRLALRRLFEVNDENLQVVAINDLTDASVLAHLFKYDSAHGKFNGEVEVLKDNGKNYLKIKGQKILVLSERDPKSLPWGQLGIDLVVECTGFFASKSGASQHLEAGAK

P---------D------------­­------------------------------------------P------------------------------------------------------

KVIISAPAGNDVKTIVYNVNCDTITEDDRILSSASCTTNALAPLVNALDKEFGIDHGFMTTIHAYTADQRLQDAPHGDLRRARAAGVNLVPSSTGAAKSIGLVVPSLTGKLDGIAIRVPV

------------------------PP----------------------------------------------------------------------------------------------

ITGSFVDLSVELKSNPSIEEINQKMREYANESFAYCDEPIVSSDIIGDRHGSIFDATLTKYIEANGKRLYKLYTWYDNEYSFVSQFVRVIRDFVQK

-----------------P----------------------------------------------PP------------------------------

**After Cleavage**

MKKIAINGFGRIGRLALRRLFEVNDENLQVVAINDLTDASVLAHLFKYDSAHGKFNGEVEVLKDNGKNYLKIKGQKILVLSERDPKSLPWGQLGIDLVVECTGFFASKSGASQHLEAGAK

P-D---D-D-D-D-------P-PPP-------------------------PP-----------PPP--------------------P---------------------------------

KVIISAPAGNDVKTIVYNVNCDTITEDDRILSSASCTTNALAPLVNALDKEFGINHGFMTTIHAYTADQRLQDAPHGDLRRARAAGVNLVPSSTGAAKSIGLVVPSLTGKLDGIAIRVPV

-------------------------P---------P--E---------------P--P--D---P-PP--XXXXXXXXXEP-D-------DDDDD-------------------------

ITGSFVDLSVELKSNPSIEEINQKMREYANESFAYCDEPIVSSDIIGDRHGSIFDATLTKYIEANGKRLYKLYTWYDNEYSFVSQFVRVIRDFVQK

-----------------P---------PPPPP-------------PPP---------------PPP-----------------PP-----P--P—

# Supplementary Data S10: Negative control for cell lysis during intact cell washing and non-specific binding to avidin agarose during affinity chromatography.

**Colony counts of *Mycoplasma hyopneumoniae* (strain 232) before and after 3 washes of intact cells with 1 × PBS on two replicates. Note: No difference in colony counts for after centrifugation replicates.**

**
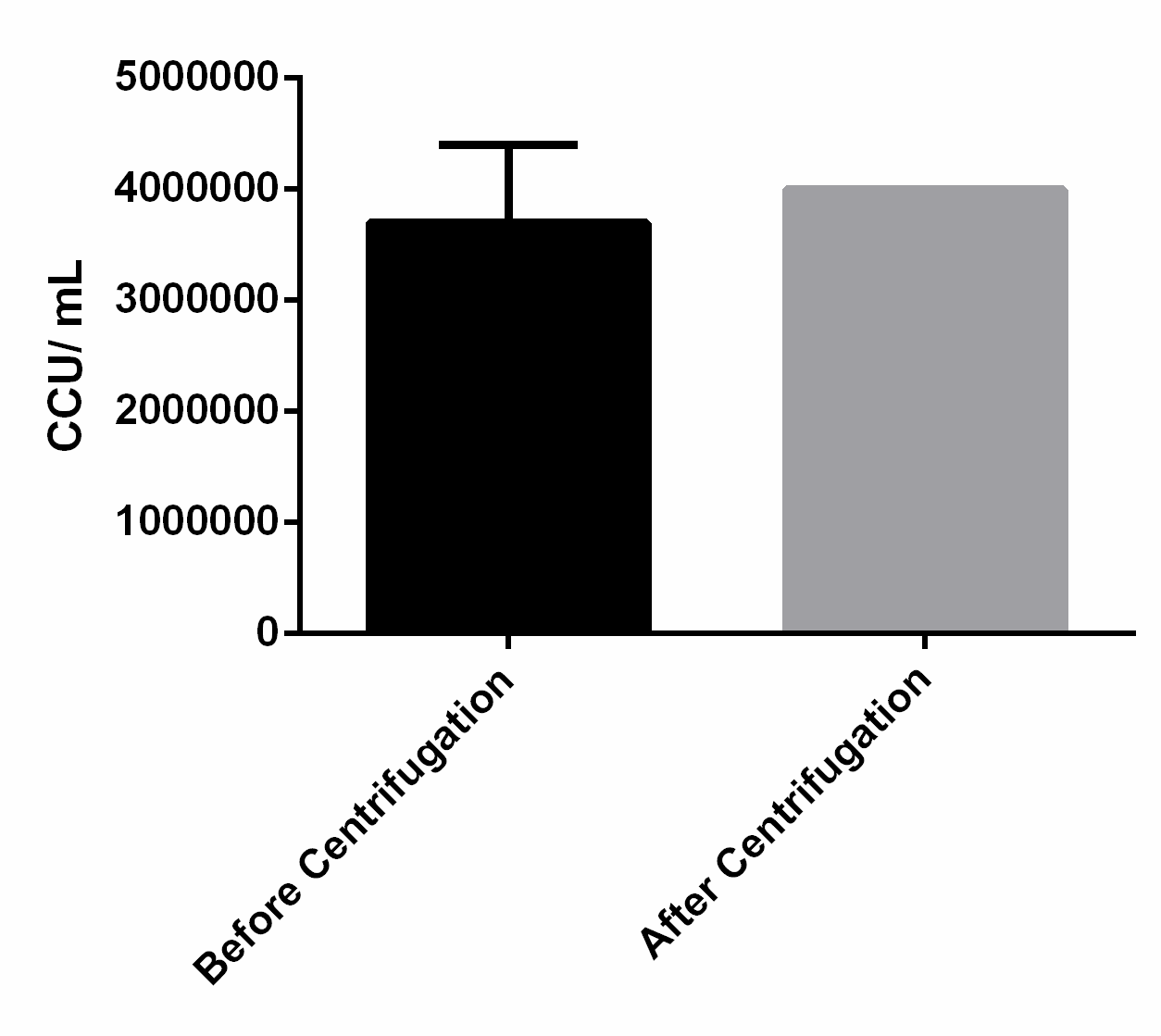
**

# Supplementary Data S11: Control gel of *Mycoplasma hyopneumoniae* lysates through avidin agarose column without host bait molecules attached. No *Mycoplasma hyopneumoniae* were detected after Wash 1.

**Lane 0: Molecular weight markers (BioRad Precision Plus Protein™ Unstained Standards)
Lane 1: *Mycoplasma hyopneumoniae* whole cell lysate
Lane 2: Blank loading control
Lane 3: Flow through 1
Lane 4: Flow through 2
Lane 5: Wash 1
Lane 6: Wash 2
Lane 7: Wash 3
Lane 8: Wash 4
Lane 9: Elution 1
Lane 10: Elution 2
Lane 11: Elution 3
Lane 12: Elution 4**

**
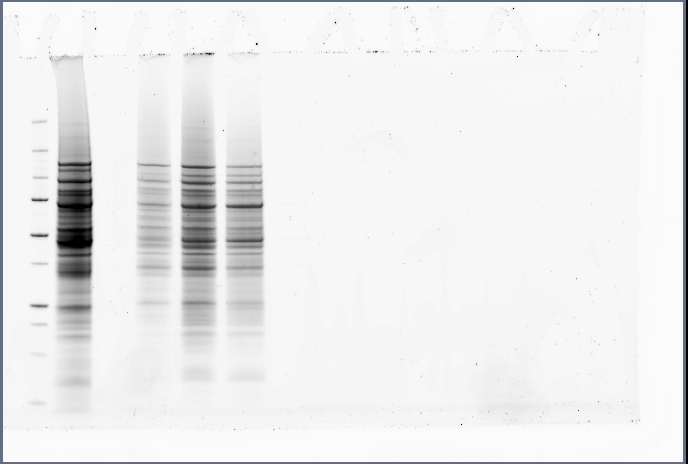
**

**250kDa**

**150kDa**

**100kDa**

**75kDa**

**50kDa**

**37kDa**

**25kDa**

**20kDa**

**15kDa**

**10kDa**

**0 1 2 3 4 5 6 7 8 9 10 11 12**
